# Charge Extraction Multilayers Enable Positive-Intrinsic-Negative Perovskite Solar Cells with Carbon Electrodes

**DOI:** 10.1021/acsenergylett.4c03403

**Published:** 2025-05-13

**Authors:** Tino Lukas, Seongrok Seo, Philippe Holzhey, Katherine Stewart, Charlie Henderson, Lukas Wagner, David Beynon, Trystan M. Watson, Ji-Seon Kim, Markus Kohlstädt, Henry J. Snaith

**Affiliations:** ◧ Department of Physics, 6396 University of Oxford, Clarendon Laboratory, Parks Road, Oxford OX1 3PU, U.K.; ‡ 28477 Fraunhofer Institute for Solar Energy Systems ISE, 79110 Freiburg, Germany; § Centre for Processable Electronics, 4615 Imperial College London, London SW7 2AZ, U.K.; ∥ Philipps-University Marburg, Renthof 7, 35032 Marburg, Germany; ⊥ SPECIFIC, College of Engineering, Swansea University, Skewen SA1 8EN, U.K.

## Abstract

Perovskite solar cells achieve high power conversion efficiencies but usually rely on vacuum-deposited metallic contacts, leading to high material costs for noble metals and stability issues for more reactive metals. Carbon-based materials offer a cost-effective and potentially more stable alternative. The vast majority of carbon-electrode PSCs use the negative-intrinsic-positive (n-i-p) or “hole-transport-layer-free” architectures. Here, we present a systematic study to assess the compatibility of “inverted”, p-i-n configuration PSC contact layers with carbon top electrodes. We identify incompatibilities between common electron transport layers and the carbon electrode deposition process and previously unobserved semiconducting properties in carbon electrodes with unique implications for charge extraction and electronic behavior. To overcome these issues, we introduce a double-layer atomic layer deposited tin oxide (SnO_2_) and Poly­(2,3-dihydrothieno-1,4-dioxin)-poly­(styrenesulfonate) (PEDOT:PSS), yielding up to 16.1% PCE and a retained 94% performance after 500 h of outdoor aging. The study is a crucial step forward for printable, metal-electrode-free, and evaporation-free perovskite PV technologies.

## Graphical abstract

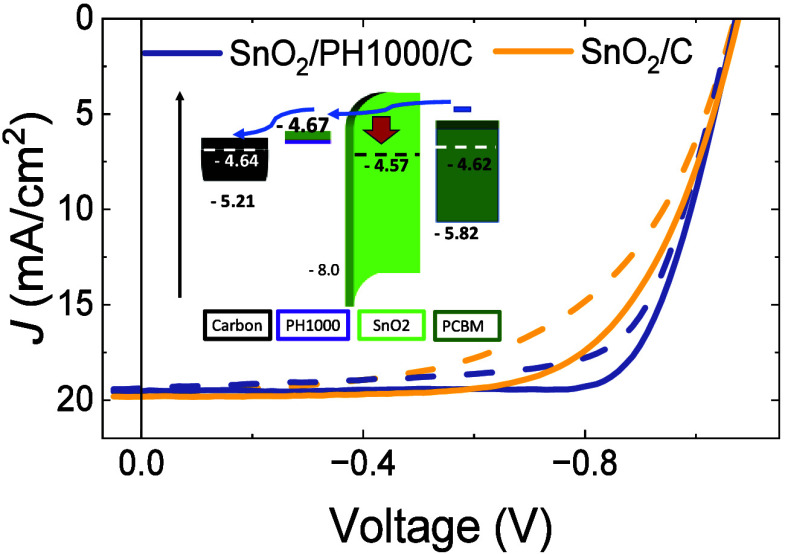

Over the past decade, perovskite solar cells (PSCs) have evolved as a promising candidate for next-generation photovoltaics due to their exceptional material properties, including solution processability, direct and tunable bandgap, high defect tolerance, and strong light absorption.^1–7^ These properties gave rise to rapid advancements in power conversion efficiencies (PCEs), now reaching over 26.2% in single junctions, outperforming most traditional photovoltaic technologies.^8^ However, PSCs can suffer from poor operational stability, with most research cells deteriorating within months. For successful commercialization, lifespans of 20–30 years are needed.^9^

Electrodes that selectively collect electrons and holes are essential for both the performance and stability of perovskite solar cells. Unlike the bottom electrodes, which are usually commercial transparent conducting oxides (TCOs) such as ITO and FTO, the terminal counter electrode is approached in various materials and processes and significantly influences device operation.^10–13^ In state-of-the-art perovskite solar cells (PSCs), noble metals are predominantly used as counter electrodes, which have been linked to long-term stability issues, including interdiffusion of halides or metal under heat and light, forming undesirable interface or charge recombination centers.^10,11,14,15^ Incorporating a buffer layer (e.g., Cr) has been reported to prevent this diffusion by promoting Cr/Au alloy and effectively immobilizing Au ions.^10,16^ Indium-based TCOs have also been successfully employed as counter electrodes and are considered to be more inert than noble metals under device operational conditions. To minimize series resistance, additional metals such as silver are often integrated as grids or fingers to boost conduction.^13^ Despite these approaches to improve stability, the scarcity of indium, commonly used in TCOs, remains a challenge for TW-scale production of solar cells.^17^ This leads researchers to explore sustainable alternatives like copper, indium-free TCOs, or carbon electrodes.^18,19^

Carbon-based electrodes are an attractive alternative since they are based on a highly abundant element.^17^ These electrodes can be produced via simple wet coating processes like slot-die, blade-coating, or screen-printing, which might bring lower material and energy use compared with thermal evaporation or sputter coating.^20–23^ Graphite-based carbon electrodes show good electrical conductivity, chemical stability, and cost-effectiveness. Due to graphite displaying covalent bonds as opposed to metallic bonds, interdiffusion of the carbon electrode with the perovskite is highly unlikely, and reactions with halides are far less favorable as compared to metallic electrodes. Being able to produce PV modules via these methods having very high throughput, in atmospheric pressure and low temperatures, remains attractive for future low-capex PV factories.^24^

Most PSC with carbon-based electrodes (C-PSC) use the n-i-p structure or the hole-transport-layer (HTL)-free architecture.^18^ However, the transport layers used in n-i-p devices, such as TiO_2_ and Spiro-OMeTAD, are frequently linked to instabilities. Within the field of PSC, the p-i-n structure has been shown to be more stable and has recently surpassed n-i-p devices in terms of absolute record efficiency.^25–27^ Despite the stability and now efficiency advantages of p-i-n cells, there is limited literature on how to effectively employ carbon electrodes. In p-i-n devices, the transport layers are significantly thinner than those in their n-i-p counterparts. This brings difficulties with the mechanical and solvent stabilities of these thin layers when carbon films are deposited on top. Furthermore, carbon is known to make good p-contact when directly processed on top of perovskite films in the hole-conductor-free architecture. These challenges may explain the comparatively low number of reports on C-PSCs on the p-i-n structure in comparison to HTL-free and n-i-p C-PSCs.

One known issue is that the solvents in the carbon paste can dissolve the thin electron transport layers (ETL). This can be inhibited by introducing a buffer layer to act as a solvent barrier. Babu et al. used a 5 nm thick layer of evaporated Cr as a buffer layer in p-i-n carbon devices, reaching steady-state efficiencies of up to 14.5%.^28^ However, an evaporated metal layer contradicts some of the perceived advantages of using a solution-processed carbon electrode.

We ensure carbon electrode process compatibility by employing an atomic layer deposited (ALD) SnO_2_ buffer layer, which prevents solvent damage from the carbon paste to the organic ETL. We reveal that the carbon electrode top surface does not behave metallically but is semiconducting in nature. This behavior is well suited for hole contact to the valence band or highest occupied molecular orbital (HOMO) of a hole-transport layer (HTL), but it is poorly matched to contact with the conduction band or lowest unoccupied molecular orbital (LUMO) or an ETL. To improve the electrical contact between the SnO_2_ and the carbon electrode, we introduce an additional semimetallic poly­(3,4-ethylenedioxythiophene) polystyrenesulfonate (PEDOT:PSS) of the type PH1000 as an interface layer preventing the formation of an energetic barrier. This approach yields higher initial performance, up to 16.1% compared to 13.7% without the PH1000 interlayer (Figure 2), and slower degradation of the C-PSCs when aged under 85 °C and 0.76 sun and outdoor conditions (Figure 3). This contributes to the wider perovskite fields as a stepping stone enabling carbon electrodes on p-i-n devices without requiring any vacuum-based deposition process.

To investigate the nature of the carbon/ETL interface, we chose the simple device stack of FTO/poly-TPD/perovskite/PCBM/carbon. Devices with this architecture and carbon electrodes showed hysteretic JV behavior and low open-circuit voltage, as displayed in Figure 1a. The resulting low maximum power point (mpp) tracked efficiencies for these devices are displayed in Figure 1b. To examine potential damage to the ETL, we increased the PCBM thickness from zero (no ETL deposited) to 30 nm (standard thickness) and then to an increased thickness of approximately 70 nm by reducing the deposition spin-speed from 2000 to 1000 rpm. Increasing the thickness results in enhanced maximum power point tracked efficiency from 0% for no ETL to less than 2% for 2000 rpm (∼30 nm) and up to 4% for 1000 rpm (∼70 nm). The current–voltage behavior of each thickness is shown in Figure S2. Additionally, we investigated the performance of devices with 70 nm of evaporated C_60_, followed by carbon paste deposition due to the expected higher resistance of this layer to solvents. These devices lead to similar behavior and performance to 1000 rpm PCBM deposition. A continuous fullerene layer of 70 nm thickness should cause a considerable series resistance in a device. However, none of the JV scans, as shown in Figure 1a and Figure S2, show resistive behavior of the expected magnitude. On the other hand, when using the same 70 nm C_60_ layer with a gold electrode, the devices show a big series resistance and close to zero percent efficiencies, as visible in Figure S3. To explore why our p-i-n carbon cells work so poorly, we imaged and scrutinized cross sections of the devices via scanning electron microscopy (SEM). In the cross section shown in Figure 1c, we cannot discern the ETL layer between the carbon and the perovskite layers. Likely, the carbon paste completely dissolved the deposited fullerene layer.

**Figure 1:**
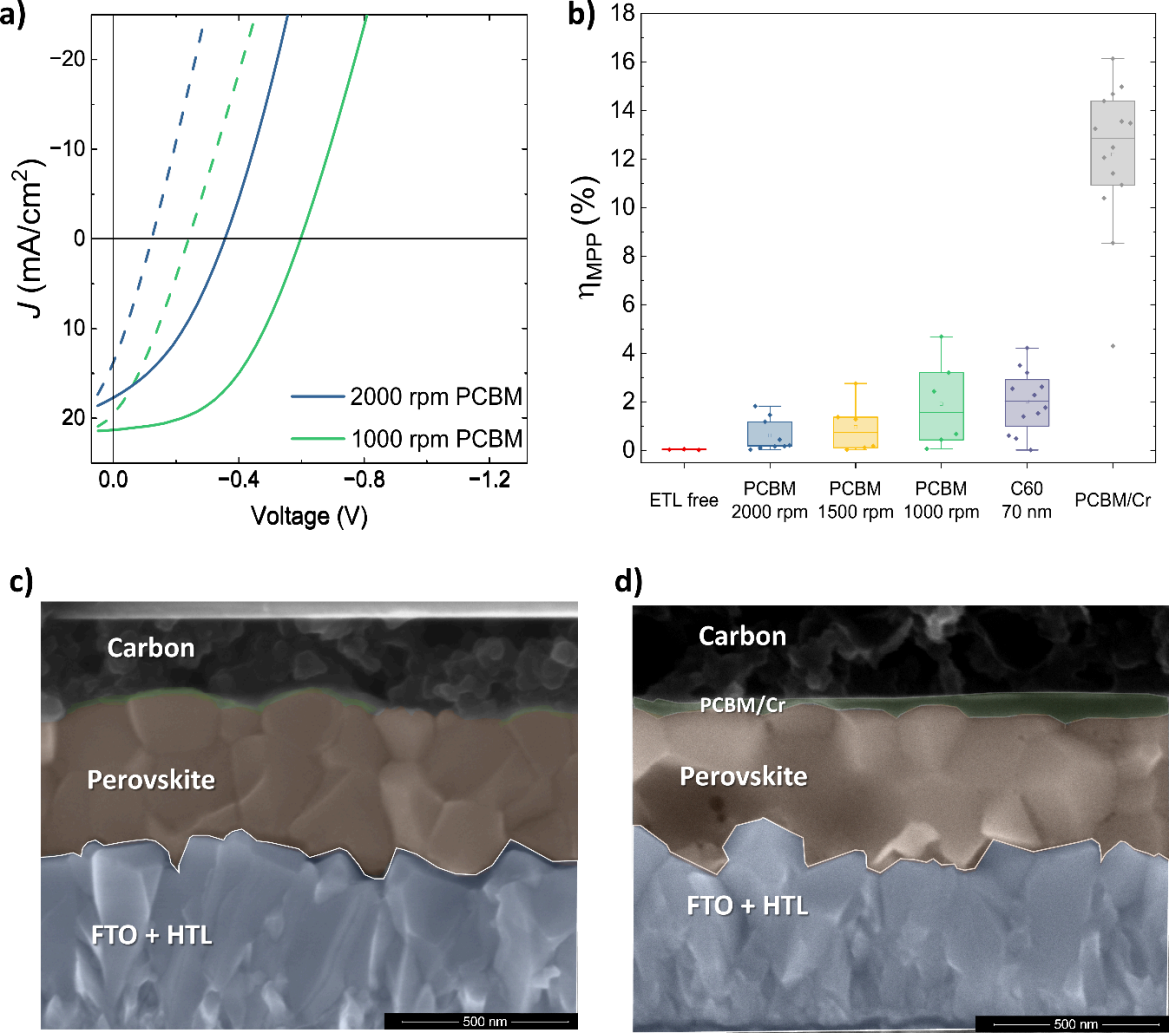
Carbon paste dissolving fullerene-based layers (a) show the JV characteristics of devices with PCBM layers deposited with different spin speeds between the perovskite absorber layer and the carbon electrode. MPP tracking efficiencies of different device types are shown in panel b. Panels c and d show SEM cross sections of a device with PCBM deposited before carbon without (c) and with (d) a 5 nm Cr buffer layer in between. The extent of FTO, perovskite, and the PCBM layer (if visible) is indicated by an artificial transparent blue, brown, and green color scheme, respectively. The unannotated images are shown in the Supporting Information (Figure S5).

Building on results reported by Babu et al., we used a 5 nm layer of evaporated Cr between the PCBM and the carbon electrode.^28^ As shown in Figure 1b, devices with such a Cr buffer layer showed high steady-state efficiencies of up to 16.2%, which is the highest reported efficiency for p-i-n perovskite cells using carbon electrodes. Once again, inspecting cross-sectional SEM images for the Cr buffer layer incorporating devices shows a distinguishable ETL layer running between the perovskite and the Cr/carbon layers (Figure 1d).

To further investigate the charge selectivity of the perovskite/PCBM/carbon layers, we fabricated transport-layer-free stacks on the ITO substrates. ITO/perovskite/carbon stacks without any transport layer showed identical JV behavior to ITO/perovskite/PCBM/carbon stacks (Figure S4). The reverse scan runs through the second quadrant, indicating a hole instead of electron selectivity at the top contact. For devices with a Cr interlayer between the PCBM and carbon, the scans were run through the fourth quadrant, showing the intended electron selectivity of the top contact. The sign change in steady-state *V*_OC_ measurements in Figure S4 confirms that trend with ITO/perovskite/carbon stacks generating n-i-p cells, with steady-state open-circuit voltages of >0.5 V under illumination and ITO/perovskite/PCBM/Cr/carbon creating p-i-n cells, with *V*_OC_s of around −0.25 V.

Because these solution-processed materials appear to be unsuitable for use with carbon pastes (SI results), we present an alternative using a layer of SnO_2_ grown by atomic layer deposition (ALD). While not a solution process, ALD is a promising high-throughput deposition technique used, for example, to deposit aluminum oxide in silicon solar technology,^29,30^ and can be adapted to ambient pressure^31^ and roll-to-roll processes. Atmospheric pressure ALD and pulsed chemical vapor deposition (CVD) SnO_2_ have been used successfully in perovskite solar cells.^32,33^ Blade-coated carbon electrodes are scalable and roll-to-roll compatible, as shown by Beynon et al.^34^ Our resulting devices, which employ an ALD deposited SnO_2_ interlayer between the PCBM and the carbon paste, exhibit power conversion efficiencies (PCEs) of around 12%, which is significantly better than those using the neat PCBM ETL layer (Figure 2a). However, they do exhibit relatively low fill factors, as compared to the Cr/carbon cells, indicating the presence of increased series resistance. To the best of our knowledge, these represent the first p-i-n carbon-electrode-based perovskite solar cells without any layer deposited in an evaporation process.

**Figure 2:**
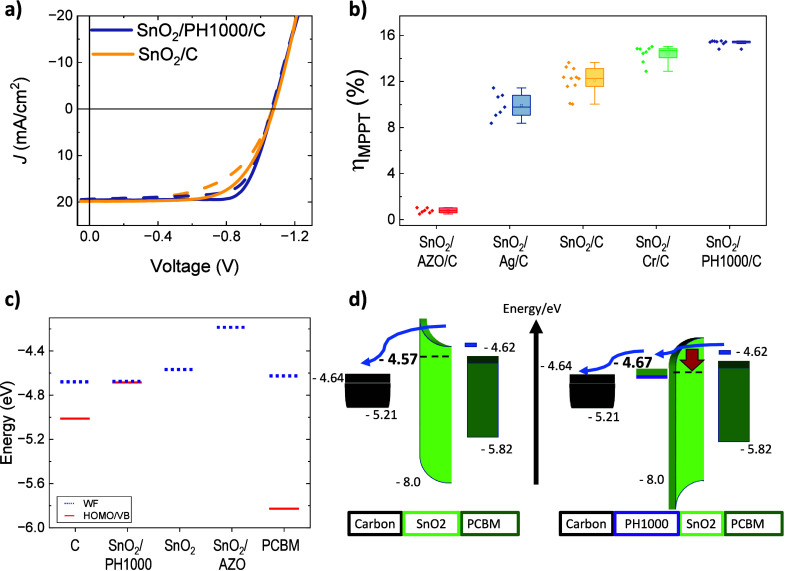
ALD SnO_2_/carbon JV scans of champion p-i-n carbon devices with and without PH1000 between the SnO_2_ layer and the carbon are shown in panel a. The maximum power point tracked performances of devices with different surface modifiers between SnO_2_ and carbon are shown in panel b. In panel c, the position of the work function (WF, blue dotted line) and the valence band maximum or highest occupied molecular orbital (VBM or HOMO, red solid line) are compared for different materials in the electron collection layer stack. Each symbol represents a set of Kelvin probe and photoelectron spectroscopy measurements on the given material. For multiple measurements, average values were taken. The sketch in panel d shows the expected effect of the PH1000 modification on charge extraction and band alignment. All values in the sketch are from our measurement results shown in panel c.

Since SnO_2_ is frequently and successfully used in contact with fullerene ETLs, we assume that the electrically deficient interface, which results in increased series resistance, is the SnO_2_/carbon interface. To further improve the electric contact between SnO_2_ and carbon, we assessed different conductive materials as thin interlayers, including evaporated silver and chromium and solution-processed PEDOT:PSS type PH1000, and show the resulting device efficiencies in Figure 2b. The metals were evaporated with 3 nm thickness, and PEDOT:PSS was spin-coated with a 1:10 dilution of the as-purchased dispersion in methanol at high spin-speeds to achieve a 10–15 nm thin layer. Devices with the PH1000 as an interface modifier showed the highest performance and best reproducibility (Figure 2b) and the highest fill factor (Figure 2a), with the median and hero cells exhibiting 12.2% ± 2.5% and 16.1%, respectively.

Because PEDOT:PSS layers are known to be conformal and dense, a possible closure of pinholes in the SnO_2_ layer was investigated with atomic force microscopy (AFM) measurements.^35^ A negligible reduction in the surface roughness from 5.8 to 5.5 nm of the SnO_2_ on perovskite was observed following the application of the PH1000. However, there were no major differences in the surface morphology of device stacks with and without PH1000 on top of the SnO_2_ ETL, and no pinholes were found in the SnO_2_ without the PH1000, suggesting interface morphology is not the main mechanism of the PH1000 device performance enhancement (Figure S15).

Since PH1000 is known to be conductive and its work function is similar to that of graphite,^36^ we tested whether a very thin spin-coated layer can significantly alter the surface work function (WF), as compared to untreated SnO_2_. With Kelvin probe (KP) measurements, we were able to detect a slight change in WF of the SnO_2_ top surface from approximately −4.57 down to −4.67 eV for SnO_2_/PH1000, as shown in Figure 2c and sketched in Figure 2d. This way, the WF of the ETL at the top surface is at a very similar energy level to the measured average work function of the carbon electrode (−4.67 and −4.64 eV, respectively), indicating close energy alignment between the two layers. Ambient-pressure photoemission spectroscopy (APS) measurements showed a valence band maximum (VBM) shift from below the detection limit for pristine SnO_2_ to −4.68 eV when the PH1000 treatment was used. The VBM of the modified charge extraction layer stack is, therefore, at the same level as its WF, indicating a metallic-like behavior of this interface. Between the carbon work function and its APS-measured HOMO on the other hand, there is a surprising difference of about 0.3 eV, indicating a semiconducting behavior of this surface (Figure 2c). All measurement data leading to the data plotted in Figure 2c are shown in Figure S14. Our measurements show that the PH1000-treated SnO_2_ achieves energy alignment with the carbon electrode at the interface, with both layers displaying work functions of around −4.7 eV. However, the metallic behavior of the SnO_2_/PH1000 layer and the semiconducting nature of the carbon surface point to an intricate interaction.

Carbon is more commonly employed as a hole-collection contact in perovskite solar cells and has been reported to exhibit hole selectivity when directly contacting the perovskite.^37,18,38,19^ Despite this charge selectivity being important for device performance, it has not been thoroughly investigated. The semiconducting top layer might block electrons from reaching the metallic bulk of the carbon electrode, while facilitating the extraction of holes. ETL-free devices showing no voltage in MPP tracking implies that pure carbon electrodes can be selective to holes only when directly contacting the perovskite and not to electrons in a planar device with HTL/perovskite/carbon (ETL-free) stacks. This is consistent with the findings presented in Figure S4 showing that an ITO/perovskite/carbon device displays n-i-p-like behavior.

The primary focus of this work is to enable the use of carbon electrodes for electron extraction in p-i-n configuration PSC while being compatible with high-throughput, nonvacuum-based manufacturing. A significant motivation for investigating carbon electrodes in perovskite solar cells was the hypothesis that they can enable higher operational stability than cells using metallic electrodes that are not gold, with the latter being prohibitively expensive and unsuitable for PV deployment.^17^ To investigate this hypothesis on our p-i-n carbon devices, we aged sealed cells under simulated sunlight at 85 °C and outdoors under real-world illumination. For the sealing, we employ a glass coverslip glued with a UV-curable epoxy resin. No edge sealant encapsulation or desiccant was used. A photograph of the substrate as mounted on a roof is displayed in Figure S13b, and further encapsulated devices are shown in Figure S22a. We note that this is not standard packaging for PV cells and modules for outdoor use, so our expectation was not to have extremely stable devices. First, we show a comparison between the SnO_2_/PH1000/carbon architecture devices and SnO_2_/carbon cells without the PH1000 modification, aged at 85 °C (Figure 3a and Figure S18). A considerable stability advantage was detected when SnO_2_ was treated with PH1000. Under this accelerated aging condition, the SnO_2_/carbon champion device power conversion efficiency (PCE) fell rapidly in performance from 13.9% measured under continuous maximum power point tracking (MPPT) to 8.0% in only 3 h of aging. The degradation was mainly due to a rapid reduction in current density and fill factor (Figure 3a and Figure S18). The champion device with PH1000 reduced only from 14.9% to 12.9% MPPT in the same time period. The SnO_2_/PH1000/carbon cells showed, after an initial burn-in, a higher performance than the untreated counterpart. There was, however, also a reduction in the fill factor for the PH1000-modified devices (Figure S13a and Figure S18). This was due to the development of “s-kinks” in the current–voltage characteristics, as visible in Figure S13b and Figure S20c–e, indicating the emergence of an energetically unfavorable contact at least one of the internal interfaces in the device. Under 0.76 sun, at 65 °C, in ambient air, encapsulated carbon electrode devices with SnO_2_ aged slower than metal-containing electrode configurations like Cr/carbon and silver (see Figure S21 and Figure S19). Under 0.76 sun and 85 °C unencapsulated conditions, SnO_2_/PH1000/carbon was even more stable than gold electrodes (Figure S20a). However, under 0.76 sun, 65 °C unencapsulated and 85 °C encapsulated gold electrodes were more stable than our carbon electrode (Figure S20a,b).

**Figure 3:**
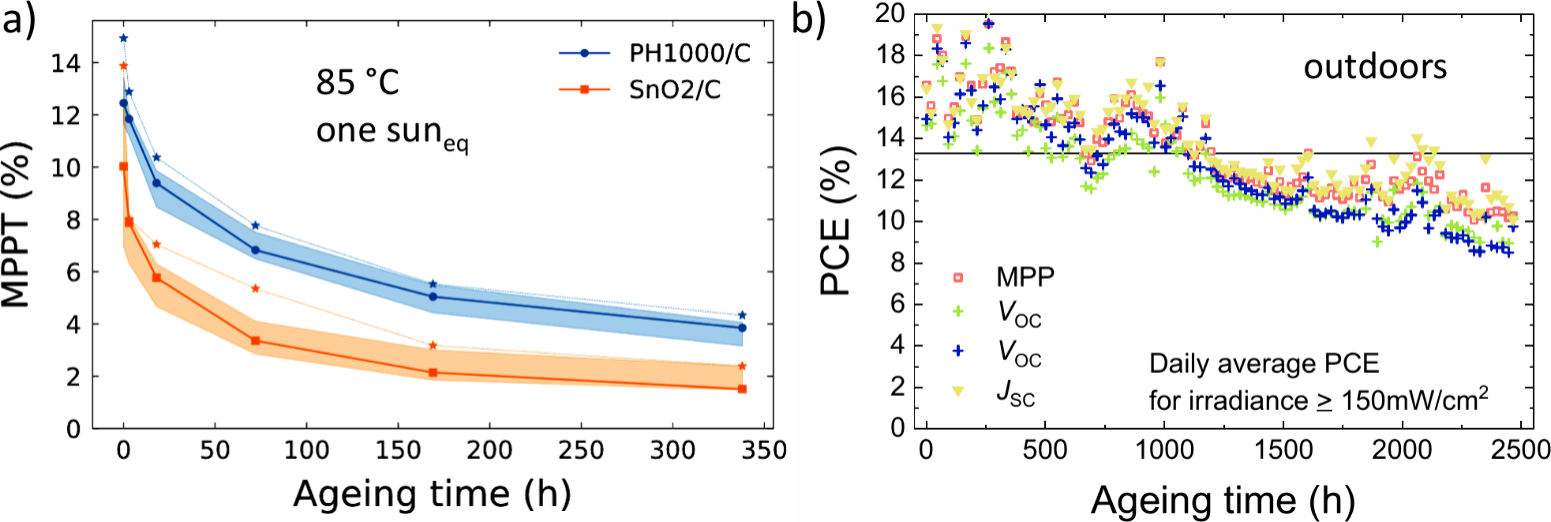
SnO_2_/carbon stability. The statistical development of maximum-power-point tracked performance upon aging under 0.76 sun equivalent illumination at 85 °C in ambient air is shown for SnO_2_/PH1000/carbon and SnO_2_/carbon in panel a. The continuous dark blue line with circles and the light orange line with squares show the median values for SnO_2_/PH1000/carbon and SnO_2_/carbon, respectively. The shaded area around this line indicates the spread from the first to third quartile. The stars above each of the shaded areas represent the respective champion values. Details of the statistics are mentioned in Figure S13. Daily average PCE values for devices aged in an outdoor setup are visible in panel b. Four SnO_2_/PH1000/carbon devices were kept under different bias conditions and periodically tested.

To investigate the aging behavior of carbon cells under real-world outdoor conditions, we mounted four solar cells with SnO_2_/PH1000/carbon electrodes in an MPP tracking system on a rooftop in Freiburg, Germany (longitude 48°), between the beginning of April and the end of May 2023 (see Methods). In Figure 3b we show the evolution of the daily average efficiencies of devices with this architecture kept at different bias conditions, including short-circuit, maximum power point, and open-circuit. All 4 devices tested were fabricated on the same glass substrate. The measured devices showed an initial PCE of between 14.6% and 16.6% at 931 W/m^2^, which is close to their last measured performance under simulated sunlight. Surprisingly, the cells degraded at a similar rate regardless of the electrical biasing conditions under which they were held. The encapsulated devices maintained ∼94% of their initial performance for about 500 h and 80% for about ∼1200 h under real operation conditions (Figure 3b). We note that the devices were exposed to ambient temperature cycles between −1 and 36 °C, with direct precipitation of up to 21 mm, as well as relative humidity between 40 and 80%. The irradiances reached values of over 1300 W/m^2^ (Figure S13d).

Treating the SnO_2_ top interface with PH1000 slows down the evolution of “s-kinks” in the current–voltage curves during aging and inferred resistive behavior under light and heat stress (Figure S20d,e). Devices with a SnO_2_/PH1000/carbon stack age more slowly than devices with BCP/Cr/carbon or BCP/Cr/Ag (Figure S21). The treatment results in a slower drop in fill factor and higher retained power output upon aging, as shown in Figure 3, Figure S18, and Figure S20. The absence of “s-kinks” in aged devices with BCP/Au instead of SnO_2_/Au (see Figure S17) suggests an energetic barrier is forming at the SnO_2_/electrode interface during combined light and heat stress. The PH1000 treatment only decelerates this degradation but does not prevent it fully. Future work on this interface is necessary to achieve desired long-term stability.

In summary, we have demonstrated that a specific combination of materials processed on top of the perovskite layer enables effective fabrication and operation of p-i-n perovskite solar cells by using carbon top electrodes. The difference in the electronic contact behavior of carbon and metal electrodes in p-i-n devices has long been overlooked and has possibly impeded the field’s progress in the direction of p-i-n devices with carbon-based electrode materials. Most common electron transport layers in p-i-n devices are either incompatible or severely damaged by the carbon blade-coating process. We have demonstrated that a dense SnO_2_ layer, together with a highly conductive PEDOT:PSS interlayer, enables an ohmic contact with a carbon electrode in a p-i-n stack. The SnO_2_ layer prevents the removal of the underlying PCBM layer, while the conductive PEDOT:PSS interlayer enables the formation of good electronic contact between the SnO_2_ and carbon and improved electron extraction in the devices. However, under long-term stress tests, the electronic nature of this contact appears to deteriorate, highlighting an important area to focus on for future research. Improving this contact may pave the way to high-performance, low-cost, and high-throughput photovoltaics. It should also enable the use of carbon electrodes on all-perovskite tandem devices, which are mostly realized in the p-i-n architecture.

## Supplementary Material



## Notes

Experimental Methods, additional figures, additional explanations and data on outdoor aging, reproducibility of the PH1000 treatment, and solution-processed alternative buffer layers (PDF)

## †

Perovskite Tandem Solar Cell Group, Helmholtz-Zentrum Berlin, Kekuléstraße 5, 12489 Berlin, Germany
