# Supplementary material for: Charge Extraction Multilayers Enable Positive-Intrinsic-Negative Perovskite Solar Cells with Carbon Electrodes
Source: ACS Energy Lett. 2025 May 13;10(6):2736–42. doi: 10.1021/acsenergylett.4c03403 (PMC12172029; doi:10.1021/acsenergylett.4c03403)
Supplement: Supplementary file 1 [file nz4c03403_si_001.pdf]

Charge Extraction Multi-layers Enable Positive-intrinsic-negative Perovskite Solar Cells with Carbon Electrodes

*Tino Lukas[a,b], Seongrok Seo[a], Philippe Holzhey[a,†], Katherine Stewart[c], Charlie Henderson[c], Lukas Wagner[d], David Beynon[e], Trystan Watson[e], Ji-Seon Kim[c], Markus Kohlstädt[b], Henry J. Snaith[a]**

[a] Department of Physics, University of Oxford, Clarendon Laboratory, Parks Road, Oxford OX1 3PU, UK

[b] Fraunhofer Institute for Solar Energy Systems ISE, 79110 Freiburg, Germany

[c] Centre for Processable Electronics, Imperial College London, London, UK

[d] Philipps-University Marburg, Renthof 7, 35032 Marburg, Germany

[e] SPECIFIC, College of Engineering, Swansea University, Skewen, UK

[†]Present address: Perovskite Tandem Solar Cell Group, Helmholtz-Zentrum Berlin, Kekuléstraße 5, 12489 Berlin, Germany

***Corresponding Author**

Henry J. Snaith Department of Physics, University of Oxford, Clarendon Laboratory, Parks Road, Oxford OX1 3PU, UK; E-mail: henry.snaith@physics.ox.ac.uk

Experimental Methods

Materials

Poly(4-butylphenyl-diphenyl-amine) (polyTPD) was purchased from 1-Material (CAS No. 472960-35-3). 2,3,5,6- Tetrafluoro-7,7,8,8-tetracyanoquinodimethane (F4-TCNQ) was purchased from Lumtec (CAS No. 29261-33-4). Lead iodide (PbI2, 99.999%, metals basis, CAS No. 10101-63-0), caesium iodide (CsI, 99.99%, CAS No. 7789-17-5), and lead bromide (PbBr2, ultra dry, 99.999%, metals basis, CAS No. 10031-22-8) were purchased from Alfa-Aesar, Thermo Fisher. Formamidinium iodide (FAI, CAS No. 879643-71-7) and carbon paste (DN-CP01) were purchased from Dyenamo AB. 1-butyl-1-methylpiperidinium tetrafluoroborate ($[BMP]^+[BF4]^-$, 99%, CAS No. 886439-34-5) was purchased from Sigma-Aldrich. [6,6]-phenyl-C61-butyric acid methyl ester (PC61BM, >99.5%, CAS No. 160848-22-6) was purchased from Solenne BV. Bathocuproine (BCP, 98%, 4733-39-5) was purchased from Xi'an Polymer Light Technology Corp. Aluminium zinc oxide (AZO, 2.5wt% in butanols, 7 nm) and SnO_2 nanoparticles were bought from Avantama AG. Tetrakis(dimethylamino)Tin (TDMASn, CAS No. 1066-77-9) for ALD and AP-CVD precursor was bought from Pegasus Chemicals. Deionised water (7732-18-5) ALD was used as the oxidiser. PEDOT:PSS dispersion in H_2O (CAS No. 155090-83-8) was purchased from Ossila. All other materials and solvents were, unless stated otherwise, purchased from Sigma-Aldrich. All materials were used as received without further treatment or purification.

Precursor solutions

If not mentioned otherwise, all solutions were prepared in a nitrogen-filled glovebox and filtered with a 22 µm PTFE filter. The hole transport layer solution was prepared by dissolving 1mg/ml polyTPD with 20 $_{wt}$% F4-TCNQ in toluene by stirring overnight at 70-80 °C. The perovskite precursor solution was prepared by stirring overnight 3.3 mg $[BMP]^+$ $[BF4]^-$, 256.2 mg CsI, 827.9 mg FAI, 2295.5 mg PbI_2, and 319.2 mg $PbBr_2$ in 3.2 ml DMF and 0.8 ml DMSO

to obtained 4 ml of 1.45 M solution of $Cs_{0.17}FA_{0.83}Pb_{1.01}I_{2.73}Br_{0.27}$ in 4:1 DMF:DMSO. PCBM solution was prepared by dissolving 20 mg/ml in 3:1 di-chlorobenzene:chlorobenzene. BCP was dissolved at a concentration of 1 mg/ml in IPA. PH1000 solution in H2O was diluted 1:10 in methanol.

Fabrication of perovskite solar cells

FTO substrates were either bought pre-patterned from Latech or etched with Zn powder and 2 M HCl to fit the electrode pattern. Devices were then scrubbed with a toothbrush with water and Fairy washing up liquid, rinsed DI water, and sonicated for 15 min in each DI water with 10% Decon90 cleaning liquid, DI water, Acetone, and IPA. Subsequently, substrates were UV ozon-treated for 15 min. PolyTPD was spin-coated dynamically at 2000 rpm for 20 s and substrates were annealed at ambient air for 7 min at 130 °C. Subsequently, all substrates were transferred into a nitrogen-filled glovebox. The perovskite layer was spin-coated with 250µL of precursor solution in a two-step programme at 1000 rpm for 5 s and 5000 rpm for 30 s. Five seconds before the end of the programme 400 µL of Anisol antisolvent were dispensed on the spinning substrate. The perovskite films were annealed for one hour at 100 °C. PCBM was spin-coated dynamically at 2000 rpm for 20 s and annealed for 5min at 100 °C. A PEIE nucleation layer was spun onto the PCBM with a solution of 0.025 wt% PEIE in IPA at a spin-speed of 5000 rpm for 30 s. The same spin-parametres were used for the tried buffer layers AZO, SnO_2 np, BCP, and PEI. All these films were tried for one minute at 100 °C.

Preparation of ALD-SnO_2 films

ALD thin film deposition was conducted by Picosun ALD (R-200). The ALD SnO_2 films were deposited at 100 °C. N_2 was used as a carrier and purging gas. The vessels for TDMASn and DI water were maintained at 70 and 20 °C, respectively. 20 nm thick SnO_2 films were achieved by 140 cycles consisting of 1.6 s of TDMASn pulse/10 s purging/1 s of water pulse/10 s of purging for a single cycle.

Preparation of the Electrodes

Carbon electrodes were blade-coated with a micrometre adjustable blade. The electrode pattern was achieved by masking with a polyethene sticker cut by Green Tree Ltd while blade-coating. Two different pastes were used throughout this work. One was bought from Dyenamo AB, used as delivered and dried with a hot air gun at a distance of 17 cm for 5 min. The other was produced by combining 1-Butanol solvent and ethylcellulose to form a 12.5% weight resin followed by mixing under high shear using a speedmixer to combine a 21.7% weight addition of Carbon made up of Graphite (Imerys Timrex) and Carbon Black (Imerys Ensaco 360G) in a 2.6:1 ratio and dried on a hotplate at 80 °C for 10 min.

We thermally evaporated metal layers at pressures below $2.0*10^{-6}$ torr.

Preparation of the PH1000 layer

Clevios™ PH 1000 PEDOT:PSS water-based dispersion was stored in a refrigerator. A spin-coating solution was prepared always freshly immediately before spin-coating by diluting the PH1000 dispersion into a 10:1 volume of methanol to PH1000. Redispersion was achieved on a vortex mixer. The layer was spin-coated dynamically, in air, with a spin speed of 4000rpm. A volume of 250µl per substrate was used. After a spin programme of 40 s the films were annealed for 10 minutes at 100°C.

Solar Simulator Measurements

Devices were measured with a Keithley under a solar LED array by wavelabs. An automated programme was used to measure the devices. First steady-state V_{OC} values were obtained by measuring at zero current for 10 s. This was followed by current-voltage sweeps in both directions through the range of 50 to -1400 mV. Sweeps were stopped if the current density exceeded 50 mA/cm^2. The dark sweeps were conducted first followed by the sweeps under illumination. Then, a 30 s MPPT was conducted using a gradient descent algorithm. Finally, a

10 s J_{SC} tracking at zero bias was conducted. This measurement protocol was for initial performance assessment as well as during ageing.

Encapsulation

Devices were encapsulated with a glass slide glued to the back side of the substrate over the active area with a UV-activated epoxy (Everlight Eversolar AB-341). The epoxy was applied to the glass slide before the substrate was placed in the correct position onto the epoxy glass slide followed by 3 minutes of UV-curing. To ensure good glass-epoxy-substrate contact and avoid edge effects, perovskite material was removed from the substrate at the edge of the encapsulation area. For the outdoor ageing pre-existing devices were used so that the perovskite removal could not be done retrospectively to ensure a good encapsulation.

Accelerated Ageing Tests

Devices were placed in a black (anodised) metal rack facing the glass side up in a closed Atlas suntest CPS Plus ageing box. The temperature of the box was set at 65 °C or 85 °C, as measured and controlled with a black temperature standard. They were illuminated with a Xenon lamp simulated AM1.5 sunlight (760 W/m^2). The temperature was automatically regulated by reducing the heat induced by the illumination with varying fan speeds. The artificial intelligence chatbot “ChatGPT” by OpenAI was used to help write Python scripts to analyse raw data of the periodic performance measurements.

Outdoor Ageing Test

Outdoor ageing tests were performed in Freiburg, Germany (48.010°N, 7.832°E, Köppen-Geiger climate zone Cfb). Devices under test were mounted to a fixed-plane measurement array with an inclination of 32°. The outdoor ageing was started on 5th April 2023 and was run for 2500 h, during which periodic reverse scan current-voltage measurements of all cells were performed in intervals of 15 min to monitor their performance over time. In between these measurements, cells were kept under open circuit conditions, short circuit conditions or under

load, tracking their maximum power point. For the performance analysis, only data from measurements performed at a global irradiance higher than 150 W/m2 (0.15 suns) were used.

Kelvin Probe Measurements

Measurements were carried out on bilayer (ITO/material) samples and partial device stacks using a KP Technology APS04 integrated Kelvin probe system using a gold-plated tip (ϕ: 2 mm). Samples were allowed to equilibrate in the dark whilst under measurement, with the work function reported being the stabilized value. Similar work function values were found when measurements were carried out in an N2, suggesting any degradation of the sample in air didn't significantly affect the results.

Ambient-pressure Photoemission Spectroscopy

Measurements were carried out on partial device stacks using the same KP Technology APS04 integrated Kelvin probe system described above. Samples were illuminated by a UV lamp fitted with a monochromator, which was scanned from low to high energy whilst the gold-plated tip was biased at +10 V and held at a position close (~ 1 mm) to the sample. The generated photocurrent (I_{ph}) was recorded for each incident photon energy and was plotted as either $\sqrt[2]{I_{ph}}$ for metallic surfaces or $\sqrt[3]{I_{ph}}$ for semiconducting surfaces. HOMO/VB levels were found by the intersection of the linear region of the square/cube-root photocurrent response and the baseline.

Scanning Electron Microscopy

Samples for cross-sections were prepared by diamond scribing the edges of substrates and mechanically cleaving it into two pieces. The break area lies within the active area of devices. The samples were tapped with conductive copper tape to ground them to the sample holder and transferred into a Hitachi S-4300 scanning electron microscope.

Atomic Force Microscopy

AFM measurements were carried out on a Park NX10 AFM system using non-contact mode with NCM-NHCR tip and Park SmartScan software. For each sample, 5 μm scan areas were measured across the substrate and compared to a larger scan to ensure a true reflection of the topography. AFM Z height images were processed and analysed using Gwyddion software.

Starting Systems and additional Current-Voltage Behaviours

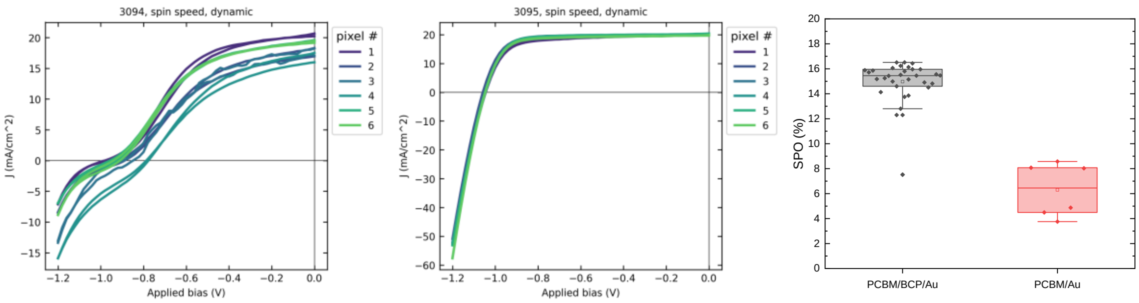

Figure S 1 A simple model system without BCP

JV forward scans for two different cell setups. The PCBM/Au champion cell without BCP hole blocking layer in (a) shows a non-ideal S-shape. On the other hand, the cell with PCBM/BCP/Au in (b) shows more ideal square-shape behaviour. (c) shows the resulting stabilized power output (SPO), held at V_{MPP} for 15 s values for both parameters.

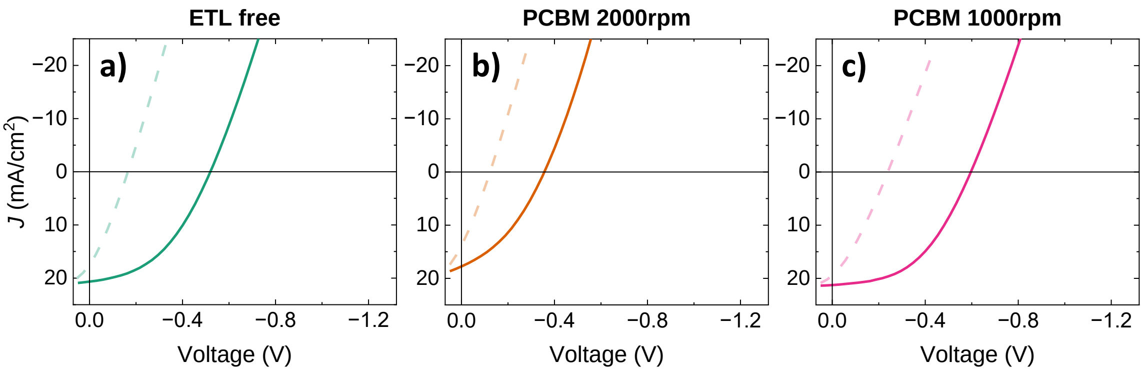

Figure S 2 JV of devices with different ETL thicknesses

(a) – (c) show JV scans of the champion devices without an ETL, PCBM deposited with a spin-speed of 2000 rpm, and PCBM deposited with a spin-speed of 1000 rpm respectively

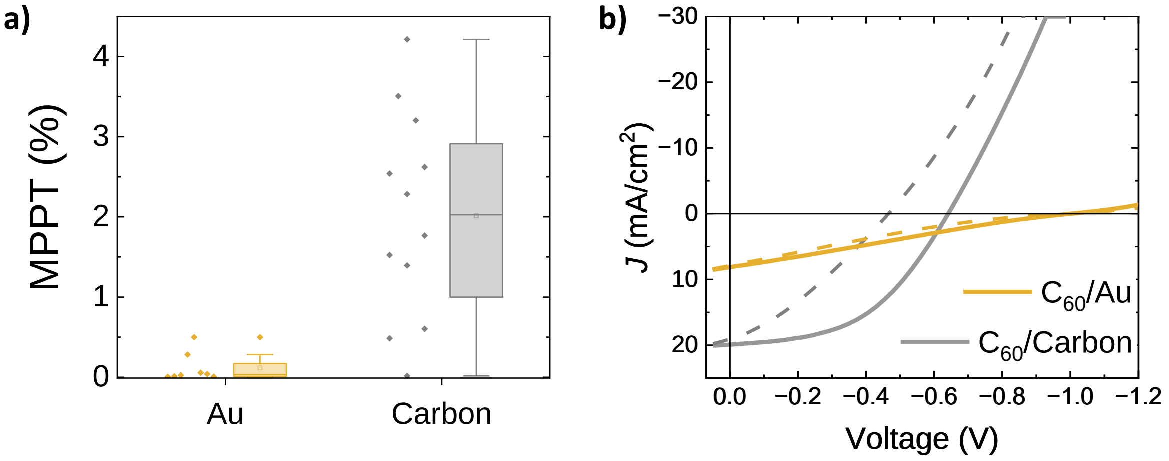

Figure S 3 Gold and Carbon devices with 70 nm evaporated C_{60}

Plot (a) shows the statistical distribution of performance via a 30s MPPT of gold and carbon devices with a 70 nm thick evaporated C_{60} layer. Characteristic JV scans of such devices are shown in (b). Devices with the thick C_{60} layer and gold electrodes showed very high series resistance resulting in a low current density and a fill factor of about 0.25. Devices with carbon electrodes reached maximum power point tracked performances of up to 4.2%. The JV scans showed high hysteresis and low V_{OC}.

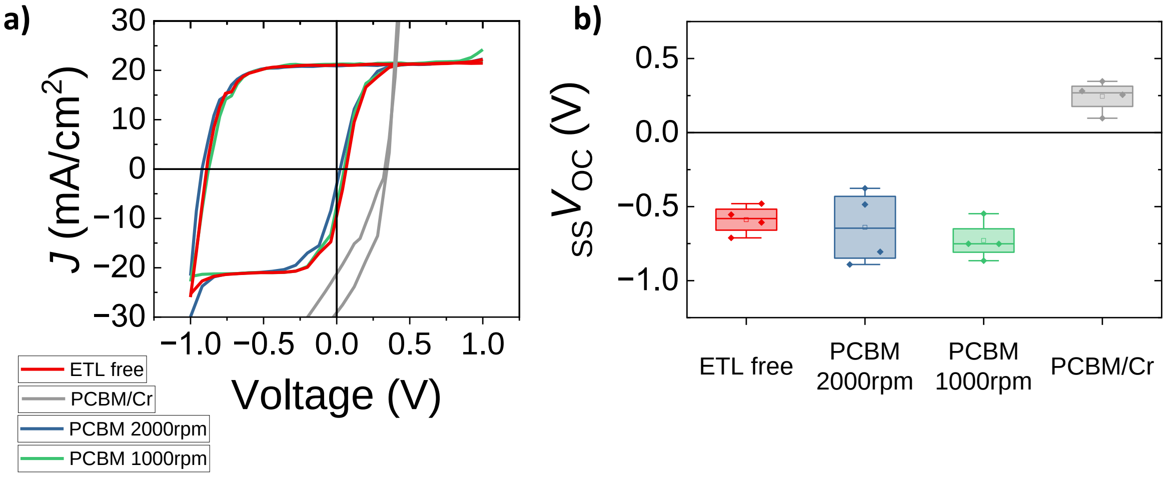

Figure S 4 Half-stack polarity of PCBM/Carbon devices

JV characteristics in (a) and steady-state open circuit voltage reached after 60 s (${}_{SS}V_{OC}$) in (b) of i-n half stacks. All half stacks were fabricated with perovskite on ITO then either directly followed by carbon depicted in red and called ETL free, ITO/perovskite/PCBM with two different spin speeds followed by carbon shown in blue and green and ITO/perovskite/Cr/Carbon displayed in grey. The ITO/perovskite/Carbon stack without any transport layer behaved very similarly in JV and ${}_{SS}V_{OC}$ to the half stacks where PCBM was deposited between perovskite and carbon. The JV curves in reverse scans run through the through the second quadrant showing n-i-p device behaviour with over 12% PCE. The forward scan runs close to the origin showing minimal p-i-n behaviour. Both scans of the devices with evaporated Cr between PCBM and carbon go through the forth quadrant showing far less hysteresis and p-i-n like behaviour. The steady-state open circuit voltage of ETL free and PCBM without Cr devices has a negative sign indicating as well n-i-p device behaviour whereas the ones with Cr buffer have a positive sign showing p-i-n like behaviour.

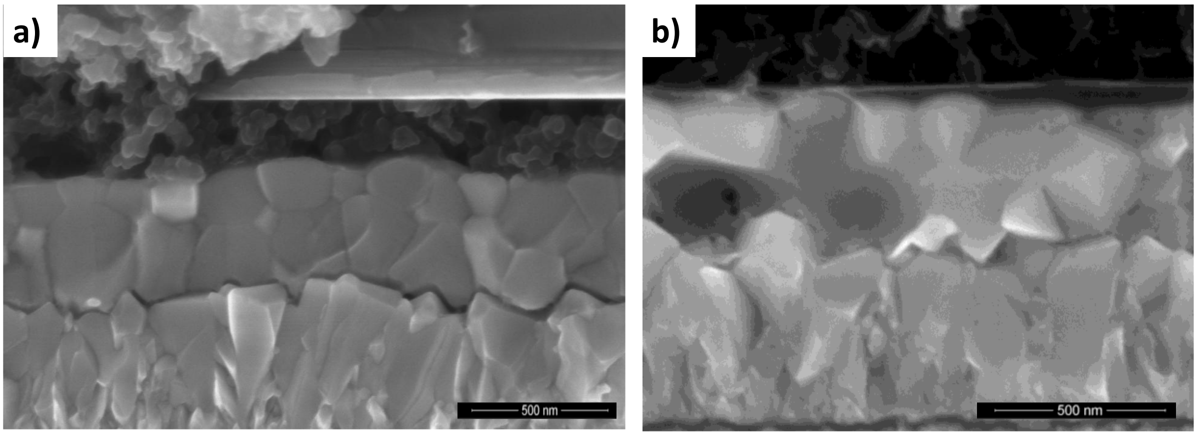

Figure S 5 Scanning Electron Microscopy (SEM) Images

The SEM cross-sections show devices with only PCBM (a) and PCBM followed by 5 nm evaporated (b) in the stack between perovskite and the blade-coated carbon electrode reapctively

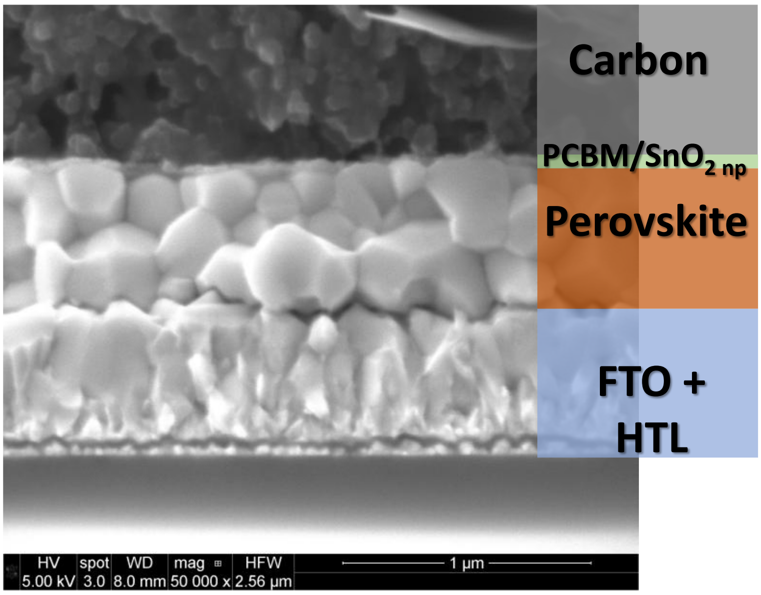

Figure S 6 SnO_2 nanoparticle (np) carbon device SEM cross-section

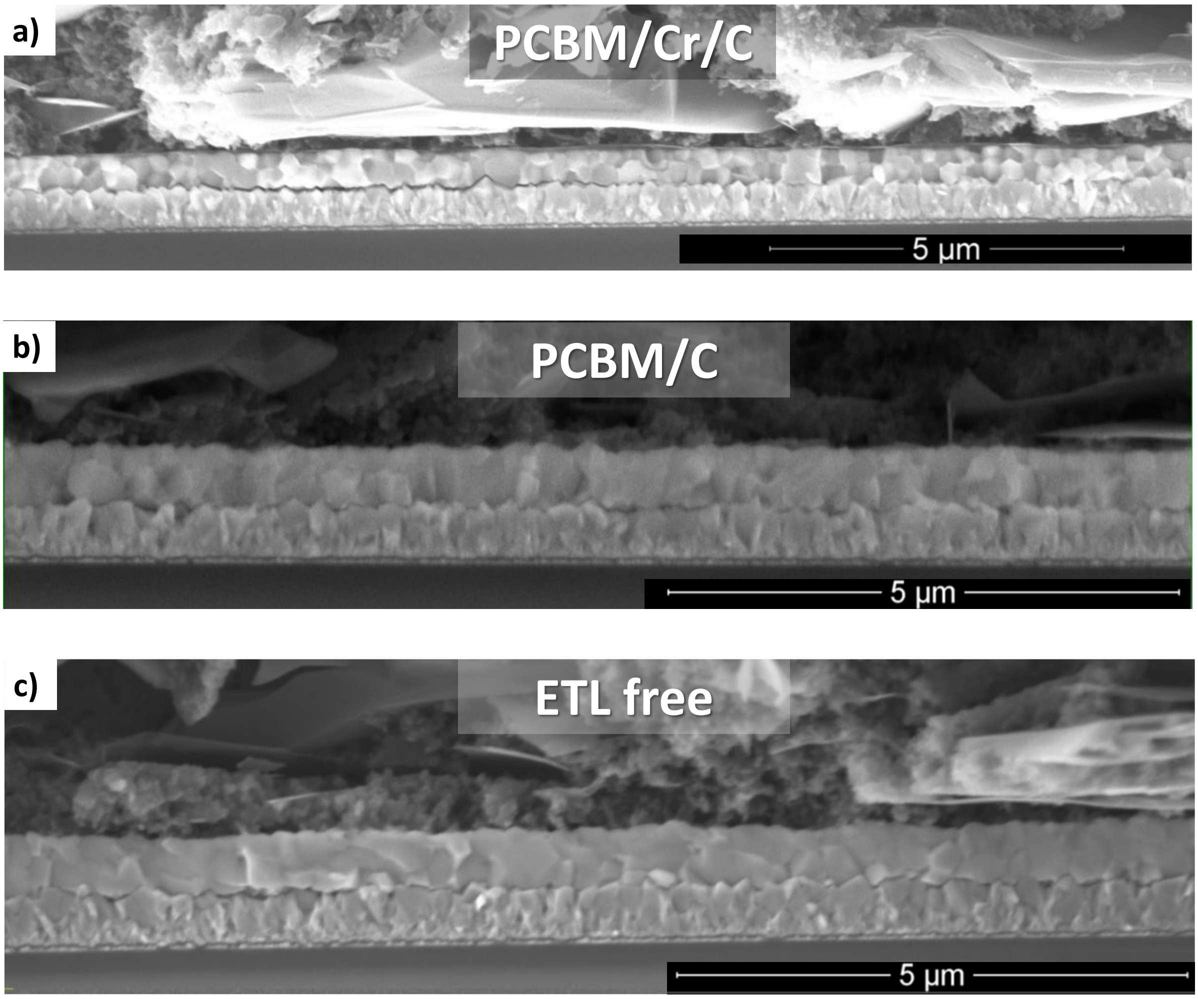

Figure S 7 PCBM carbon device cross-sections

The SEM cross-sections (a) – (c) show devices with PCBM and 5 nm Cr, only PCBM, and not ETL in the stack between perovskite and the blade-coated carbon electrode

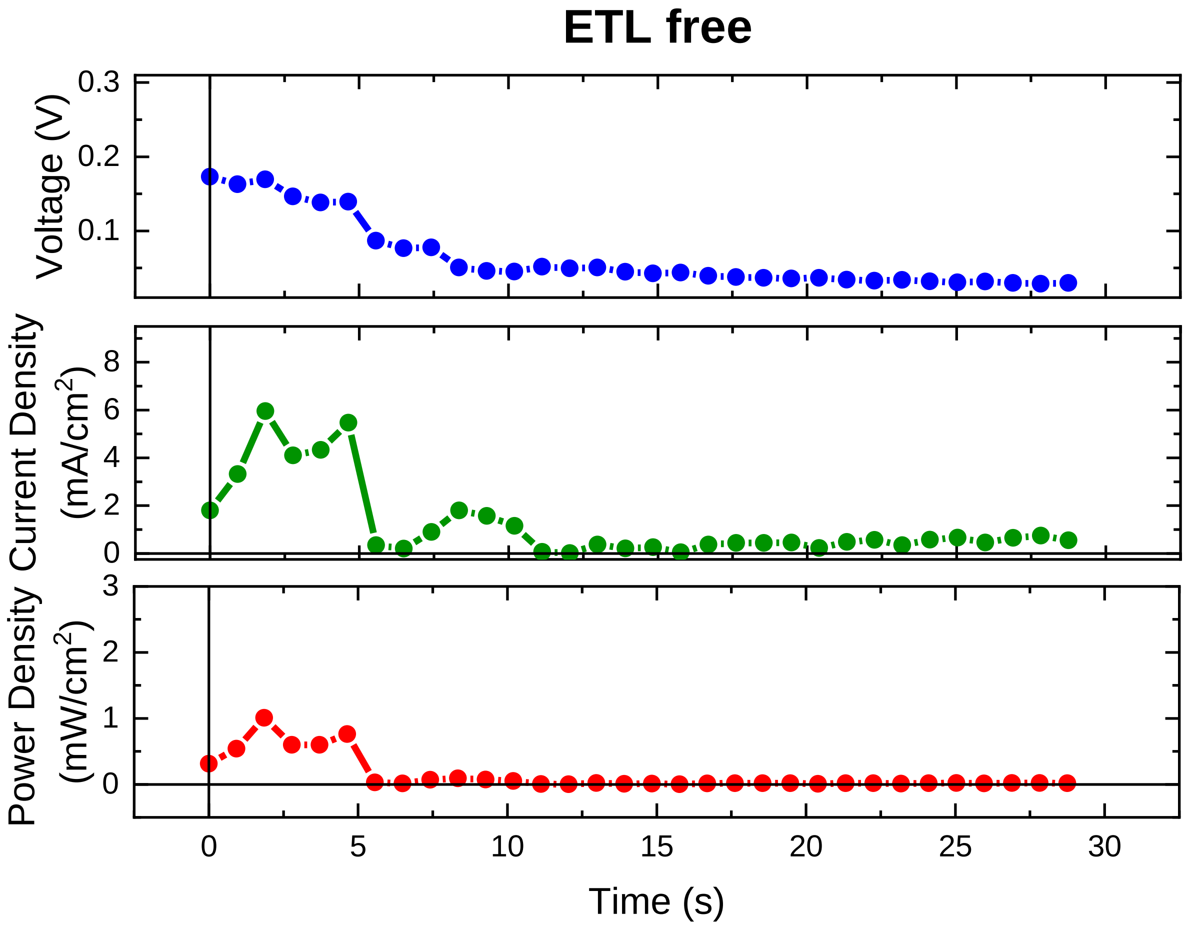

Figure S 8 Maximum power point tracking of an electron transport layer free device

Representative characteristics of the development of current density behaviour when the applied voltage is changed with a gradient descent algorithm to keep the device at its maximum power point. All electron transport-layer-free devices have a perovskite layer sandwiched directly between a hole transport layer and a carbon electrode. Such devices showed an initial performance and current density voltage behaviour to devices with a PCBM layer between perovskite and carbon. But within less than 30s their performance falls to less than 0.1% in all cases.

Solutions processed buffer layer alternatives

To obtain a fully solution-processed PSC, we investigated a selection of solution-processed layers that are typically used in p-i-n devices, to see if any of them can be a substitute for evaporated Cr. These include bathocuproine (BCP), polyethylimide (PEI), zirconium acetylacetonate (ZrAcAc), and SnO_2 nanoparticles, which were all processed on top of the PCBM layer, before carbon-paste coating. We expected these commonly used layers to work

because the work function of graphite is reported to be close to the work functions of metal electrodes successfully used with the same materials. We show, however, that a direct replacement of metal electrodes with blade-coated carbon does not yield high-performing devices with any of the mentioned solution process layers. Even though all these materials show good contact with metal (Figure S 9), none of them display characteristics of good contact formation with the carbon electrodes.

We show that replacing the evaporated Cr layers with standard solution-processed polymers, large molecules and metal oxide nanoparticles does not lead to the desired contact with the carbon electrode. Based on the existing literature we explored materials that have been shown to work well in stable inverted solar cells between a fullerene-based layer and a metal electrode. First the metal oxide nanoparticles, tin oxide[22,47–50] and aluminium zinc oxide (AZO) [51,52], secondly the polymer Polyethylenimine (PEI),[53,54] and last the large molecules zirconium acetylacetonate (ZrAcac),[55,56] and Bathocuproine (BCP),[44] and a cross-linkable fullerene derivative[57,58] called PCBCB provided by a collaborator. As shown in Figure S 9a,b. all these buffer layers contact different with carbon and metal electrodes. While they lead to high maximum power point tracked performance when used with metal electrodes they cause poor rectification in the dark and poor MPPT performance in devices with carbon electrodes. JV curves for carbon devices with some of the materials are shown in Figure S 9c. The JV characteristics with carbon devices show high series resistance and interface contact issues for all the materials. A 5 nm thick layer of evaporated Cr alleviates these contact issues completely for BCP as visible in Figure S 9a,b. Devices with the Cr nano layer under a bulk carbon electrode performed similarly to gold devices.

Layers of these materials, like the SnO_2 nanoparticles, are shown in the SEM cross-section in Figure S 6, after blade-coating the carbon electrodes on top of them, suggesting that these materials remain intact after coating with carbon, but introduce severe resistive barriers.

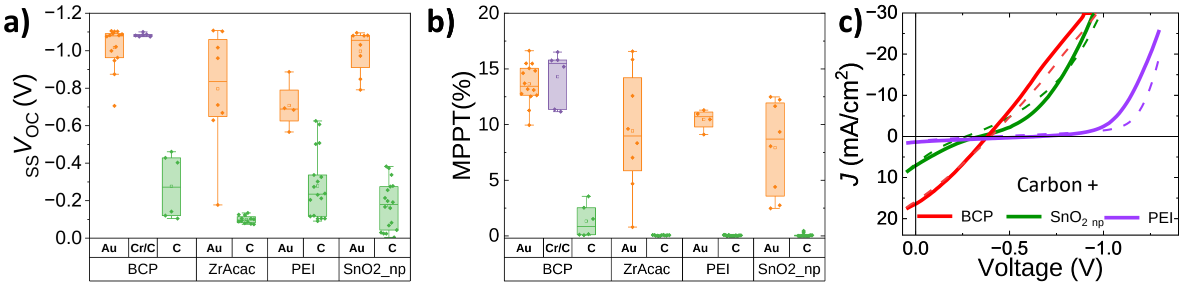

Figure S 9 Buffer layers

(a) and (b) show the MPPT performance and open circuit voltage of devices containing different layers between the PCBM ETL and the gold and carbon electrode. Gold device data is plotted in orange and carbon device data in green. For BCP there are also devices with 5 nm evaporated Cr under the carbon electrode. The JV behaviour of the best of three of the layers is shown in (c).

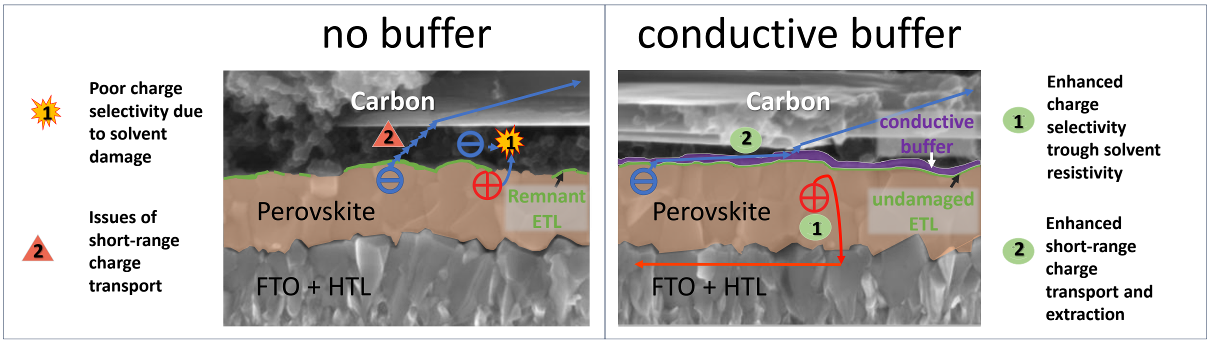

Figure S 10 Carbon devices without and without a buffer layer

The coloured SEM cross-sections above show on the left-hand side how a device without a buffer layer loses performance due to solvent damage to the ETL and hindered charge transfer to graphite flakes. The right-hand side exemplifies how a device with a buffer layer is able to perform due to good charge selectivity and extraction.

Reproducibility of PH1000 treatment

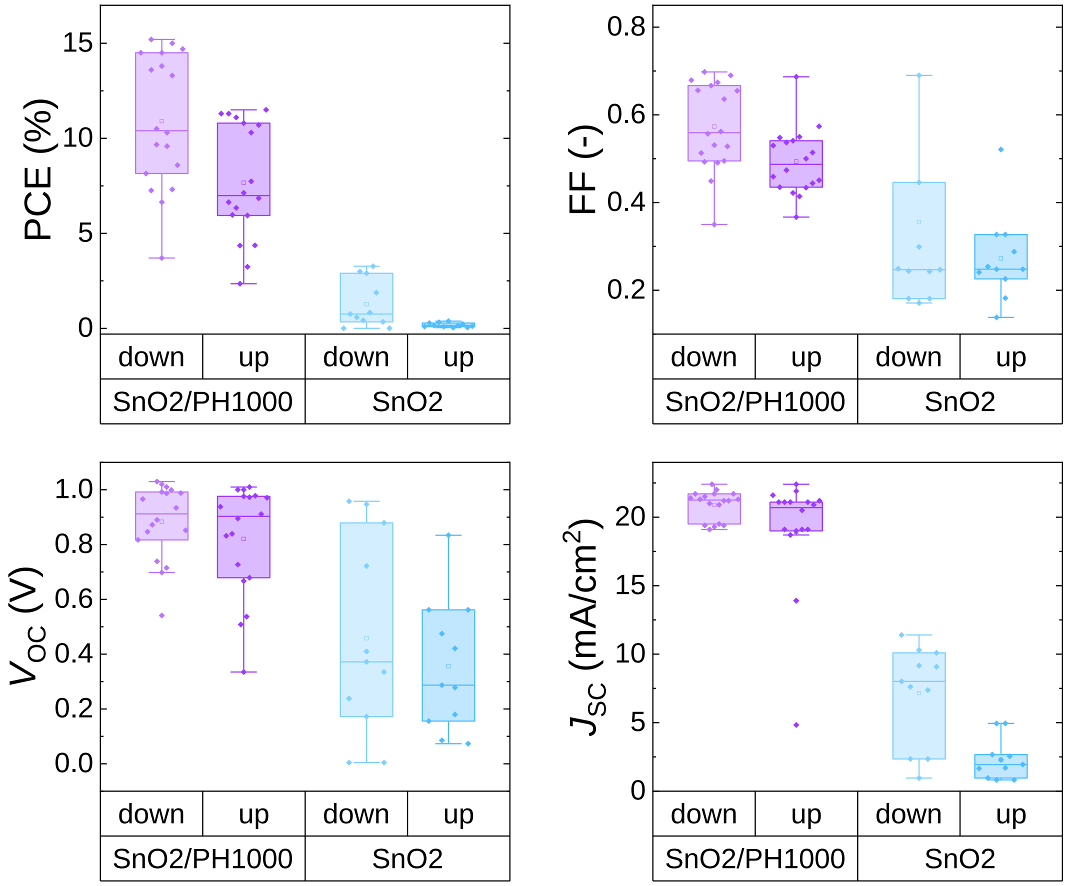

Figure S 11 Transferability of PH1000 treatment of SnO_2

Device parameters for blade-printed carbon devices with an SnO_2 ALD layer with and without PH1000 are shown. The shown parameters a retrieved from current voltage scans, devices have an active area of 0.0676 cm^2 and the used perovskite is a triple cation double halide. A GEMStar ALD system was used for the SnO_2 layer.

We show the general validity of this trend by producing SnO_2/Carbon and SnO_2/PH1000/Carbon devices in a different lab, with a different ALD system, on a different perovskite and with a different carbon paste. As shown in Figure S 11, the difference between treated and untreated SnO_2 was even larger when a GEMStar ALD system and slightly lower process temperatures were used. Untreated devices reached a maximum PCE of 3.3% and devices with the PH1000 interlayer reached up to 15.2%. This finding is a possible explanation for previous reports of a bad interface between ALD SnO_2 and carbon by Babu et al.[30]

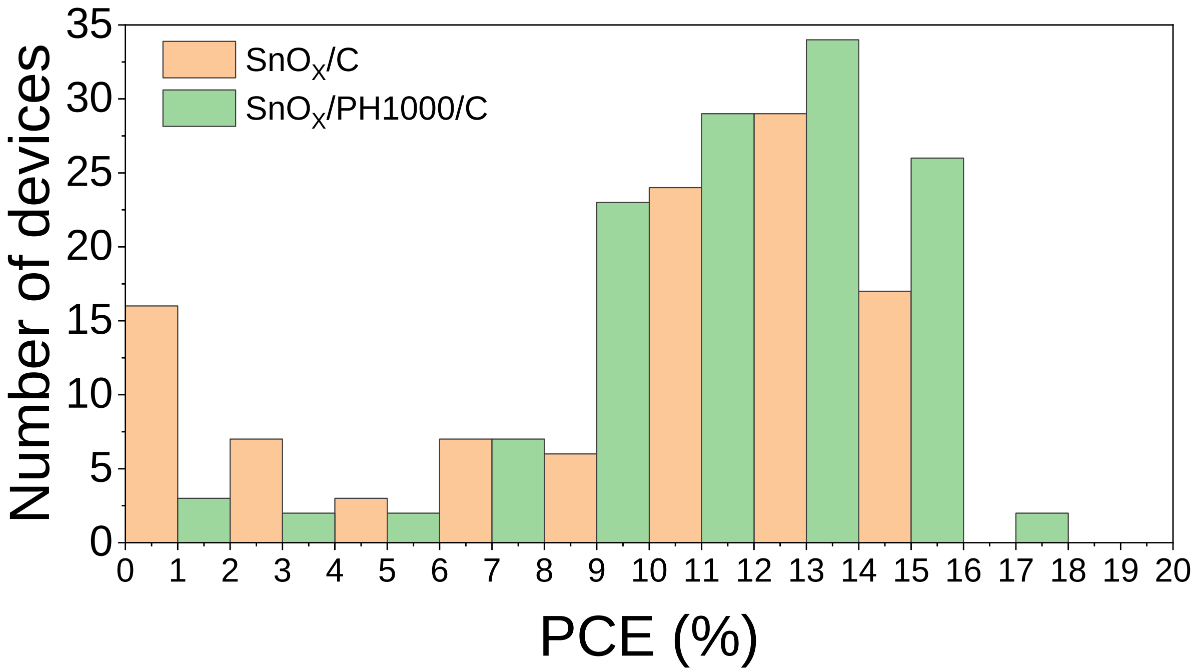

Figure S 12 multiple batch statistics of SnO_2/Carbon with and without PH1000

The number of p-i-n devices finished with SnO_2/Carbon and SnO_2/PH1000/Carbon performing in 2% performance pockets are shown. The devices were fabricated over one and a half years in two different laboratories.

We found that the performance of devices with SnO_2/Carbon depends strongly on the ALD setup and parameters used. This could also explain why Babu et al.[30] report this material combination not to work. The use of the PH1000 layer increases the batch-to-batch as well as the lab-to-lab reproducibility. Note the larger number of devices without PH1000 performing between 0 and 2% as shown in Figure S 12. None of the devices not containing the PH1000 performs between 16 and 18%.

Outdoor Ageing

In Figure S 13a the development of different performance parameters for SnO_2/PH1000/Carbon devices aged in an outdoor setup is shown. The J_{SC} values in red fluctuate a lot throughout the measurement due to different irradiances between the days, but overall did not degrade over time significantly. The V_{OC} stayed stable in this ageing study while the FF decreased rapidly causing the overall performance to drop. A photo of the device on and measurement rack after 500 h of ageing is displayed in (b). The legend of the colourmap of (c) and (d) goes from dark blue for pristine devices to dark red for devices aged 2500 h. As visible

in (c) there is a large spread in V_{OC} for the low irradiance for aged devices. Performance measurements below 150 W/m^2 were judged as unreliable and were not included in the efficiency statistics. Note the small decrease in V_{OC} over the ageing time of only about 50mV at higher irradiances. Graph (d) shows that the efficiency of the devices is on average higher at lower irradiances.

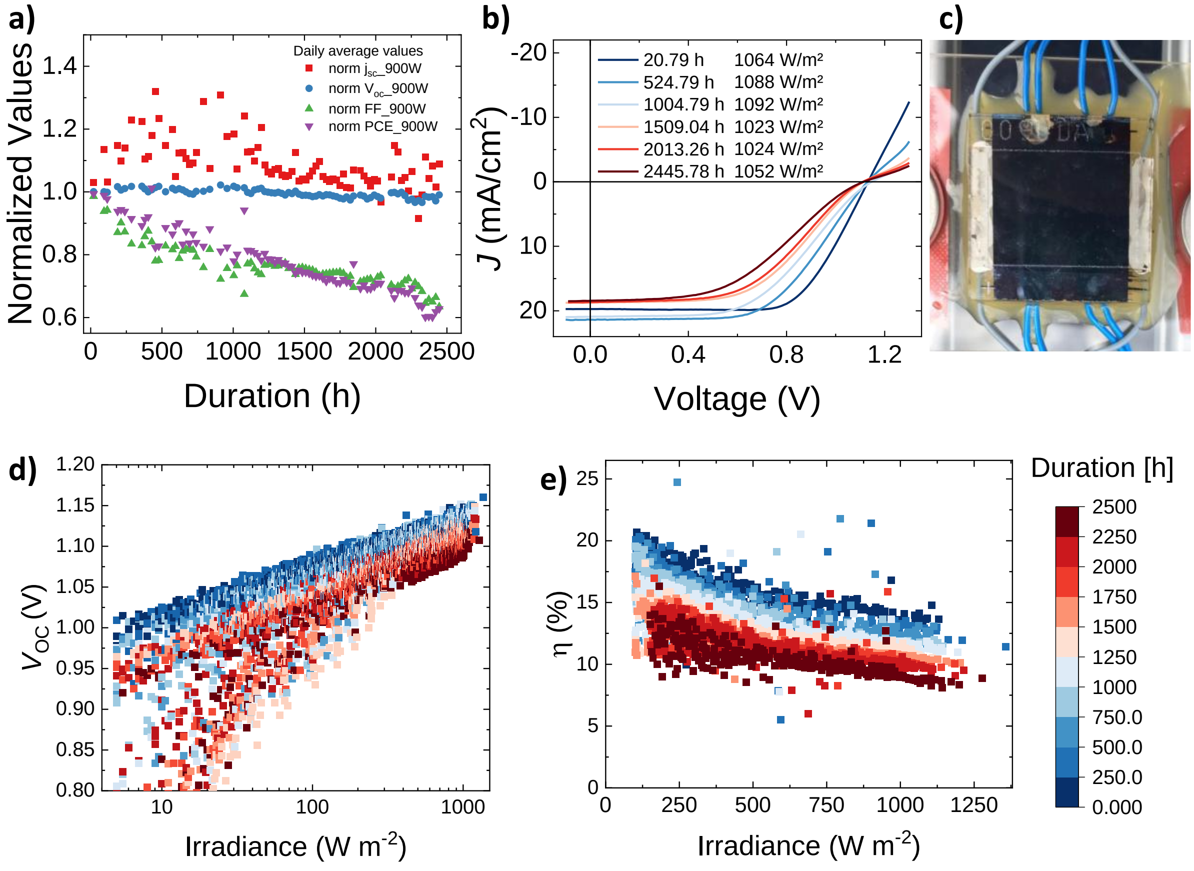

Figure S 13 SnO_2/Carbon stability

The relative development of performance parameters for devices aged outdoors are visible in (a). The corresponding JV scans for different times of ageing are shown in (b). A photo of the ageing device on the setup is displayed in (c). The open circuit voltage values and efficiencies for different irradiance values and their change over ageing time are plotted in (d) and es) respectively.

Kelvin Probe and AFM

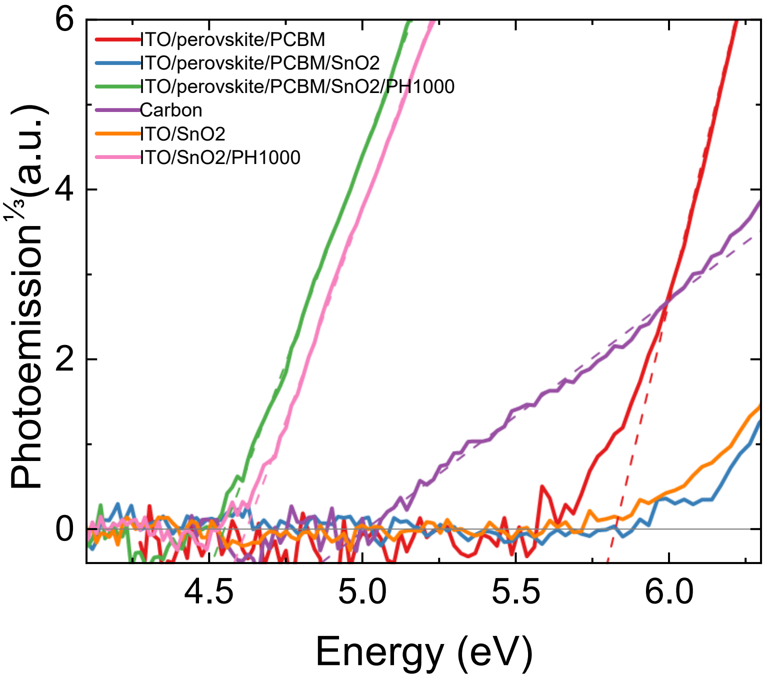

Figure S 14 Ambient-Pressure Photoemission Spectroscopy results

The Ambient-Pressure Photoemission Spectroscopy measurement results for the results in Figure 2 are shown.

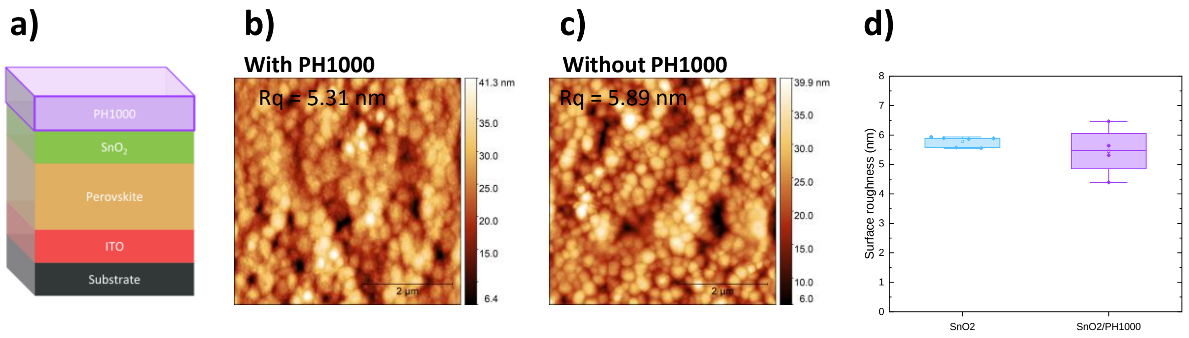

Figure S 15 AFM comparison results

(a) shows the schematic of prepared samples with PH1000 on top of an ITO/perovskite/SnO_2 samples. A representative scan for such a sample is shown in (b). A sample of the same kind of sample without the PH1000 is shown in (c) and a comparison of surface roughness values for both examples is shown in (d).

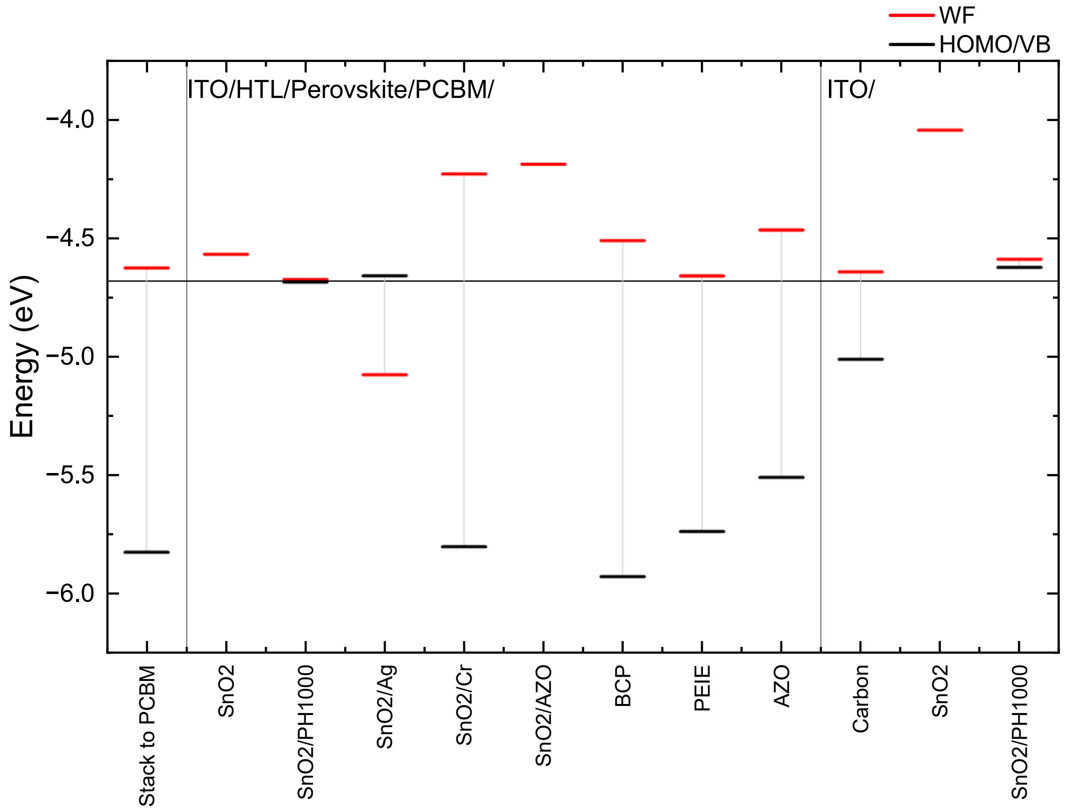

Figure S 16 Overview Kelvin Probe and Ambient Photoemission Spectroscopy results

Energy levels measured on different stacks are shown. Work function (WF) values were measured with a Kelvin Probe (KP) and the highest occupied molecular orbital (HOMO) or Valence Band maximum (VB) was measured with Ambient Photoemission Spectroscopy (APS). Left-hand side samples were measured on perovskite solar cell p-i-n stacks up until the PCBM electron transport layer. Righthand side samples were measured on grounded ITO substrates. The sample IDs are printed on top. Samples without a shown HOMO/VB level had their HOMO/VB outside (deeper) than the detector limit of about -6.0 eV. The SnO_2/Ag sample shows abnormal behaviour likely resulting from degrading while being measured.

Details Ageing Studies

Given the material properties of carbon, we would expect slower ageing compared to Au electrodes as it has also been reported widely.[19,59,60] Surprisingly, at 65 °C, one sun, in ambient air, and unencapsulated we observe the opposite (Figure S 20a). Gold is, however, too expensive for industrial applications and carbon electrodes still offer a stability advantage over other affordable electrode materials like silver (Figure S 21). Further, with increasing the temperature from 65 °C to 85 °C under the same light ambient air ageing conditions the gold electrodes pick up in ageing speed and show a similar but even quicker ageing path compared

to carbon electrodes (Figure S 20a, c-e). This stresses the need to age at higher temperatures because of characteristic ageing behaviours of gold being reported to only show above 70 °C.[10]

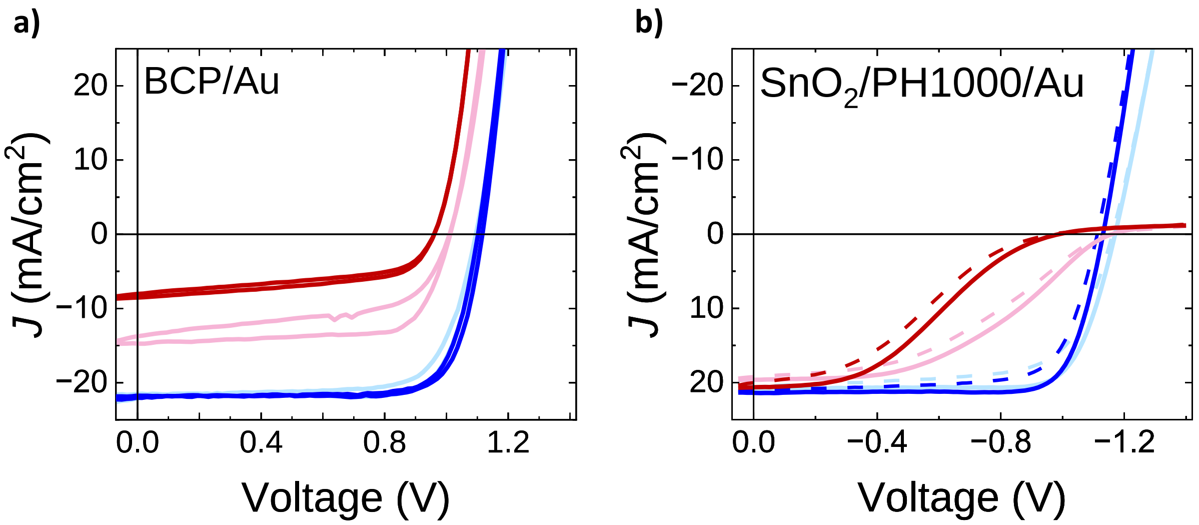

Figure S 17 BCP versus SnO_2/Au ageing behaviour

The qualitative differences between JV curve development upon ageing are shown for polyTPD/$FA_{0.83}Cs_{0.17}PbI_{2.7}Br_{0.3}$/PCBM devices finished with BCP/Au in (a) and finished with SnO_2/PH1000/Au in (b). Devices were aged for different times under different but comparable conditions of one sun illumination in ambient air at elevated temperatures above 50 °C. BCP/Au devices age usually through a loss in current density while SnO_2 devices lose fill-factor because of the development of s-kinks. This generally holds for a wide range of light-temperature ageing conditions.

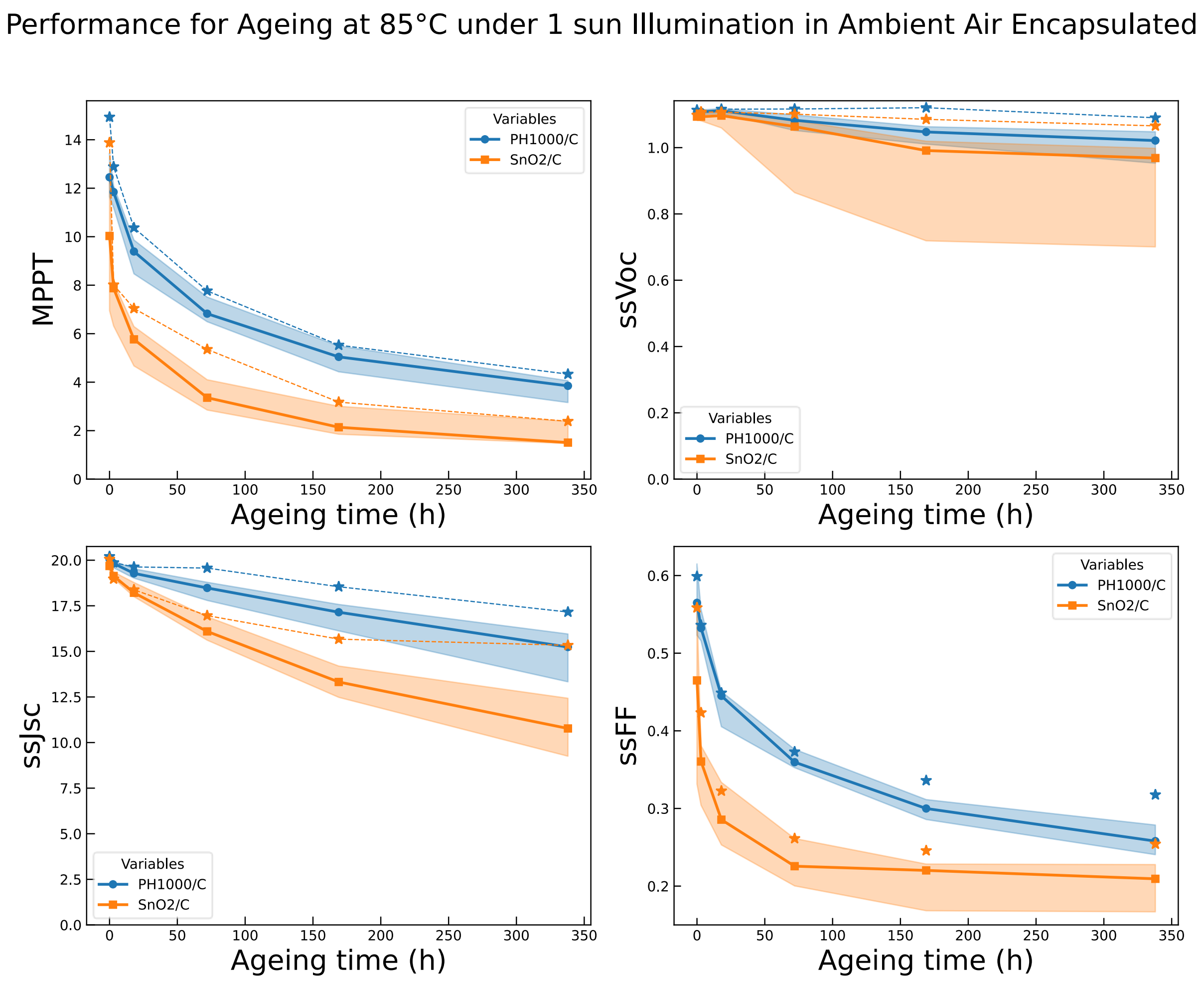

Figure S 18 Stability of SnO_2 compared with SnO_2/PH1000 buffer

The statistical development of maximum power point tracked performance parameters upon ageing under one sun equivalent illumination at 85 °C in ambient air is shown. The continuous blue line with circles and the orange line with squares show the median values for SnO_2/PH1000/Carbon and SnO_2/Carbon respectively. The shaded area around this line indicates the spread from the first to the third quartile. The stars above each of the shaded areas represent the respective champion values. For the SnO_2/Carbon 13 devices finished with these two layers were aged encapsulated. 11 devices of this category were excluded because of their initial performance before ageing being too low (< 5% MPPT). For the SnO_2/PH1000/Carbon 20 devices finished with these two layers were aged encapsulated. Four devices of this category were excluded because of their initial performance being too low (< 5% MPPT). All aged devices' active area is 0.25 cm^2.

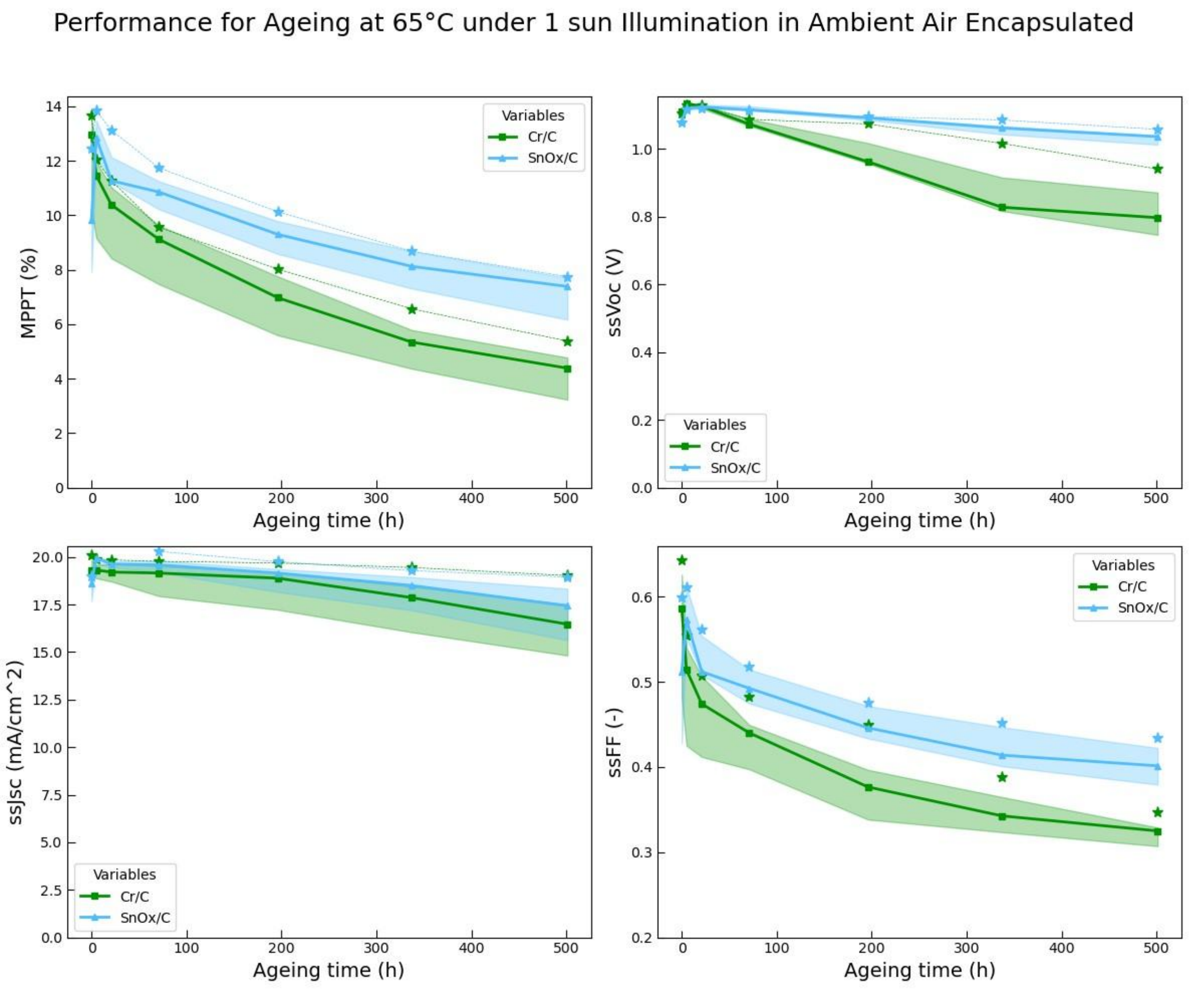

Figure S 19 Stability of SnO_2 buffer compared with Cr

The graph displays the ageing behaviour of PSC using two different electrode configurations, Cr/Carbon and SnO_2/C. Maximum power tracking (MPPT) was utilized to obtain the performance values. The median performance of six 0.25 cm^2 and two 1.00 cm^2 Cr/Carbon devices is depicted by green squares, and a solid green line is used to represent the median values. Similarly, the median performance of nine 0.25 cm^2 and one 1.00 cm^2 SnO_2/Carbon devices of the same active area is represented by blue triangles, with a solid blue line fitted to these values. Four SnO_2/Carbon devices were excluded from the statistic because of performance below 5% before starting or the ageing. Shaded areas of the same colours are plotted above and around the solid median lines, representing the extent of first and third quartiles, respectively. Notably, the Cr/Carbon devices exhibit significant initial degradation due to loss of fill factor and development of s-kinks. The SnO_2/Carbon cells demonstrate an improvement during the first five hours of ageing due to improved fill factor and current density. Over the curse of 500 h the Cr/Carbon showed faster degradation compared to the SnO_2/Carbon devices mainly due to a strong loss in open circuit voltage. All devices were glass-glass encapsulated with UV-cured glue. Ageing was performed under one sun equivalent illumination by a pulsed halogen light bulb at 65 °C in ambient air.

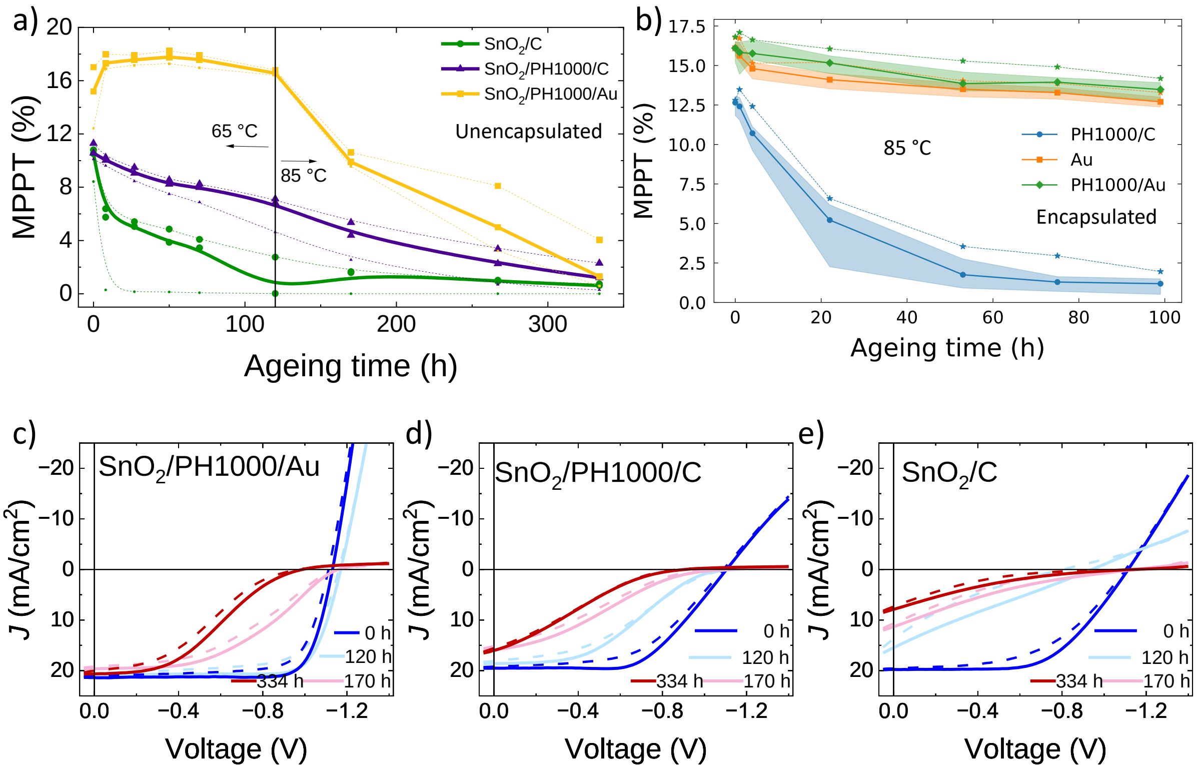

Figure S 20 Gold Carbon ageing comparison

In (a) a comparison of the ageing behaviour of p-i-n perovskite solar cell devices finished with SnO_2/C, SnO_2/PH1000/C, and SnO_2/PH1000/Au is shown. They are represented by green circles, purple triangles, and yellow squares respectively. The middle symbols connected by a solid line represent the median and the symbols above and below connected by dashed lines mark the first and third quartiles. Devices were aged unencapsulated in ambient air under 1 sun illumination. The temperature was kept for 120 h and 65 °C and then increased to 85 °C. The ageing behaviour of devices completed with SnO_2/Au, SnO_2/PH1000/Au, and SnO_2/PH1000/Carbon is displayed in (b). The medians for each parameter are shown in orange squares, green rhomboids, and blue circles respectively. The shaded areas in the same colour around the median line show the extent from first to third quartile and the star symbols above those shaded areas are the champion device performance. Current-voltage (JV) characteristics of the champion devices of the ageing study in (a) are shown in (c) – (e). A scan for the ageing times of 0 h, 120 h, 170 h, and 334 h are displayed from dark blue to dark red.

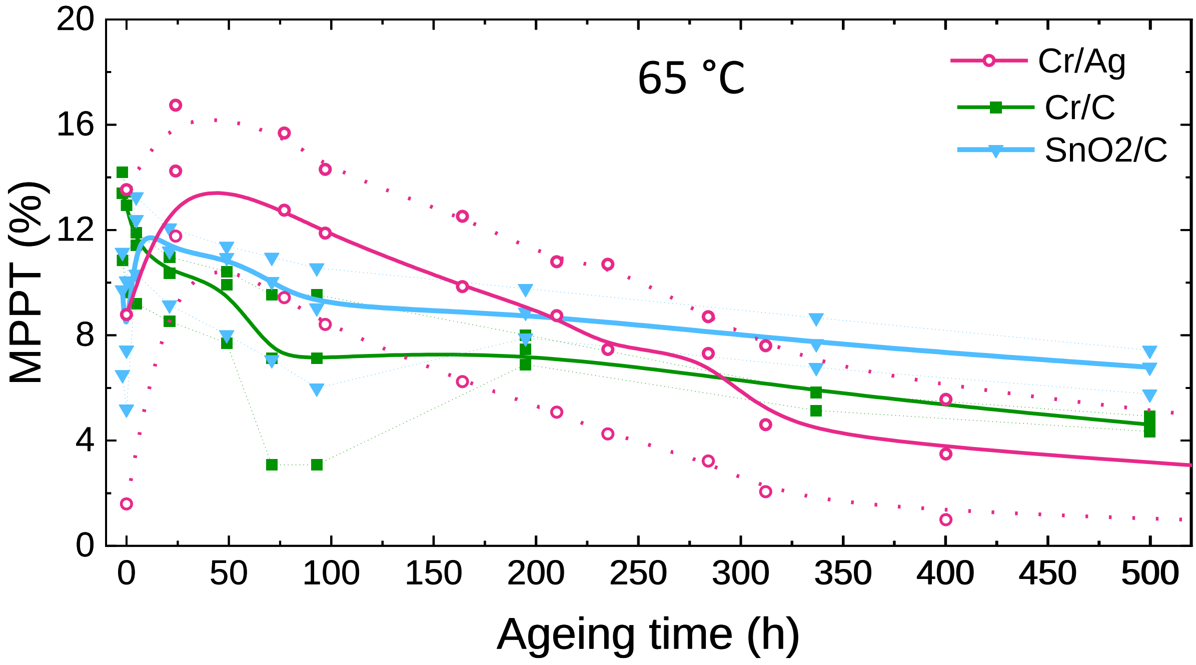

Figure S 21 Carbon versus metal stability

The graph displays the ageing behaviour of PSC out of two ageing studies comparing three different electrode configurations, Cr/Ag, Cr/Carbon and SnO_2/C. Maximum power tracking (MPPT) was utilized to obtain the performance values. Green squares depict the median performance of six 0.25 cm^2 Cr/Carbon devices, and a solid green line represents a b-spline fitted to the median values. Similarly, the median performance of 12 SnO_2/Carbon devices of the same active area is represented by blue triangles, with a solid blue line fitted to these values. Pink circles represent the performance of 16 Cr/Ag devices. Dotted lines of the same colours and symbols are plotted above and below the solid lines, representing the first and third quartiles, respectively. Notably, the Cr/Carbon devices exhibit significant initial degradation, while the Cr/Ag and SnO_2/Carbon cells demonstrate an improvement during the first hours of ageing. The Cr/Ag devices show after their initial increase a quicker degradation than the carbon counterparts. All devices were aged under one sun equivalent illumination by a pulsed halogen light bulb at 65°C in ambient air.

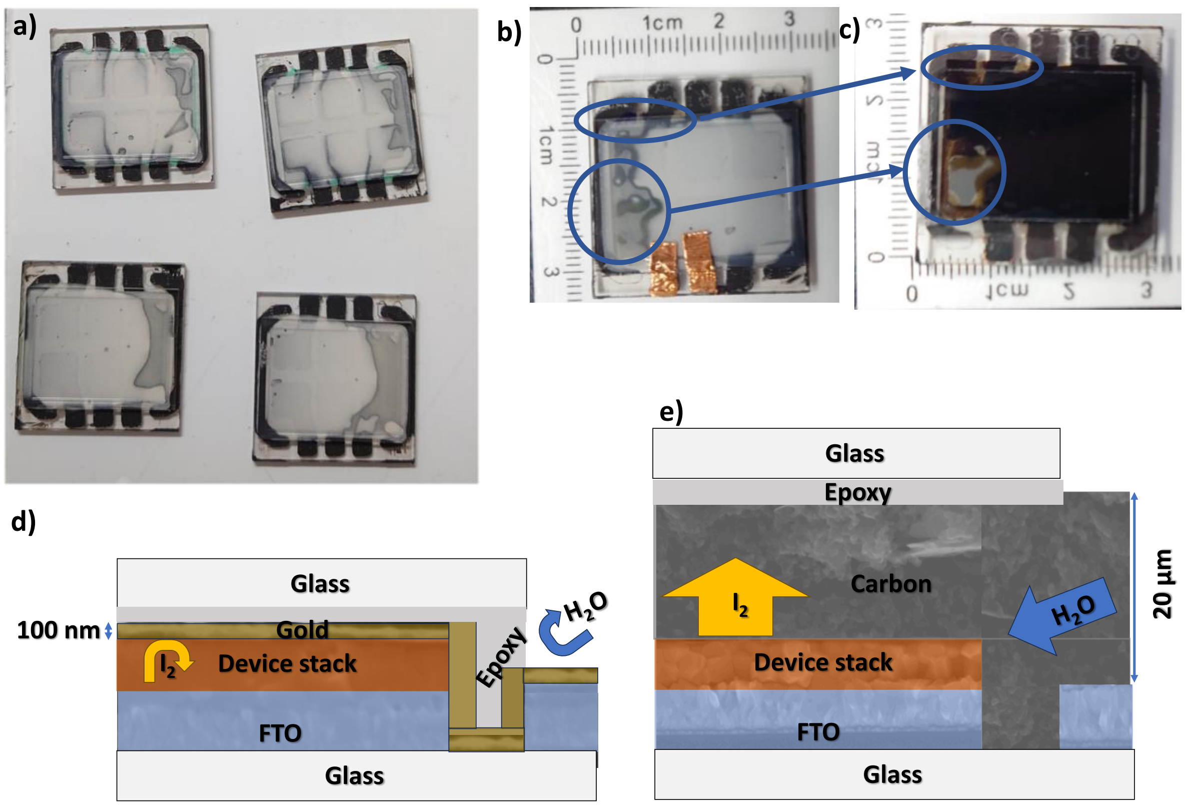

Figure S 22 Carbon Encapsulation

Problems with encapsulating devices with a carbon back electrode are shown. Devices with decorated epoxy on the back side are shown in (a). A closeup is displayed in (b). In (c) the same devices as in (b) has on the front side visible signs of degradation in the same areas where there is decorated epoxy on the backside. The anticipated schematics (not to scale) of an encapsulated device with a gold electrode are visible in (d). Problems with porous carbon electrodes when the same encapsulation is used are shown in (e).

Author information

Corresponding Author

Henry J. Snaith - Department of Physics, University of Oxford, Clarendon Laboratory, Parks Road, Oxford OX1 3PU, UK; E-mail: henry.snaith@physics.ox.ac.uk

Authors

Tino Lukas - Department of Physics, University of Oxford, Clarendon Laboratory, Parks Road, Oxford OX1 3PU, UK; Fraunhofer Institute for Solar Energy Systems ISE, 79110 Freiburg, Germany; https://orcid.org/0000-0003-0277-4838

Seongrok Seo – Department of Physics, University of Oxford, Clarendon Laboratory, Parks Road, Oxford OX1 3PU, UK

Philippe Holzhey – Department of Physics, University of Oxford, Clarendon Laboratory, Parks Road, Oxford OX1 3PU, UK; Present address: Perovskite Tandem Solar Cell Group, Helmholtz-Zentrum Berlin,Kekuléstraße 5, 12489 Berlin, Germany

Katherine Stewart – Centre for Processable Electronics, Imperial College London, London, UK

Charlie Henderson – Centre for Processable Electronics, Imperial College London, London, UK

Lukas Wagner – Philipps-University Marburg, Renthof 7, 35032 Marburg, Germany

David Beynon – SPECIFIC, College of Engineering, Swansea University, Skewen, UK

Trystan Watson – SPECIFIC, College of Engineering, Swansea University, Skewen, UK

Ji-Seon Kim – Centre for Processable Electronics, Imperial College London, London, UK

Markus Kohlstädt - Fraunhofer Institute for Solar Energy Systems ISE, 79110 Freiburg, Germany; Freiburg Materials Research Center FMF, University of Freiburg, 79104 Freiburg, Germany
